# Oxygen-Vacancy-Controlled Band-Gap Engineering in Fe/Co Co-doped La-Modified Bismuth Titanate: Integrated Experimental and First-Principles Analysis

**DOI:** 10.1021/acsomega.6c07692

**Published:** 2026-09-08

**Authors:** Mahmoud S. Alkathy, Danilo P. Kuritza, Lais Conservan Nogueira, Anibal Thiago Bezerra, Person Pereira Neves, Vitor F. Barbosa, Amatalkareem M. A. Al-Jezbi, Flavio Paulo Milton, Ricardo Pereira Bonini, Rodrigo A. R. Carvalho, Rafael Alves Lozano, Ivair Aparecido dos Santos, Alexandre José Gualdi, Marcio Daldin Teodoro, Fabio L. Zabotto, Valmor R. Mastelaro, Manuel H. Lente, José A. Eiras

**Affiliations:** † Physics Department, Institute of Exact Sciences, Federal University of Alfenas-MG, Alfenas, Minas Gerais 37133-840, Brazil; ‡ Federal University Paraná—Jandaia do Sul, Jandaia do Sul, Paraná 86900-000, Brazil; § Physics Department, Institute of Exact Sciences−Universidade Federal de Sao Carlos (UFSCar), São Carlos, São Paulo 13565-905, Brazil; ∥ Center for Environment and Sustainable Energy, Al-Razi University, Sana’a, Yemen; ⊥ Department of Physics, State University of Maringa, Avenida Colombo, 5790, Maringá, Paraná 87020-900, Brazil; # Institute of Physics of the University of São Paulo, São Carlos, São Paulo 13566-590, Brazil; ∇ Institute of Science and Technology, 67828 Federal University of São Paulo, São José dos Campos, São Paulo 12231-280, Brazil

## Abstract

Layered Aurivillius ferroelectrics offer a route to multifunctional band-gap engineering, yet the role of oxygen vacancies in band-edge reconstruction in Fe/Co-modified compositions remains poorly resolved. Here, oxygen annealing of Bi_3.25_La_0.75_Ti_2.60_(Fe_0.50_,Co_0.50_)_0.40_O_12_ (BLFC4) ceramics widens the optical band gap by 63%, from 1.35 eV (as-sintered) to 2.20 eV after 10 h of treatment, accompanied by a 5-fold drop in non-lattice oxygen (22.11% → 4.12%) and a reduction in Ti^3+^ from 42.68 to 10.47%, as quantified by X-ray photoelectron spectroscopy. Concurrently, X-ray diffraction reveals a decrease in octahedral tilt from 19° to 4°, while Raman spectroscopy shows up to 74% narrowing of B-site vibrational modes, together signaling systematic defect healing accompanied by a visible color change from black to light gray. Density functional theory rationalizes this behavior through a site-dependent vacancy mechanism: vacancies adjacent to Co-centered octahedra collapse the band gap toward a near-metallic state by introducing Co 3d states at the Fermi level, whereas vacancies near Fe leave the wide semiconducting gap intact. These combined results identify oxygen vacancy concentration rather than cation co-doping alone as the dominant lever controlling band-gap width, providing a quantitative, mechanistic framework for engineering Aurivillius ferroelectrics toward optoelectronic and photovoltaic applications.

## Graphical abstract

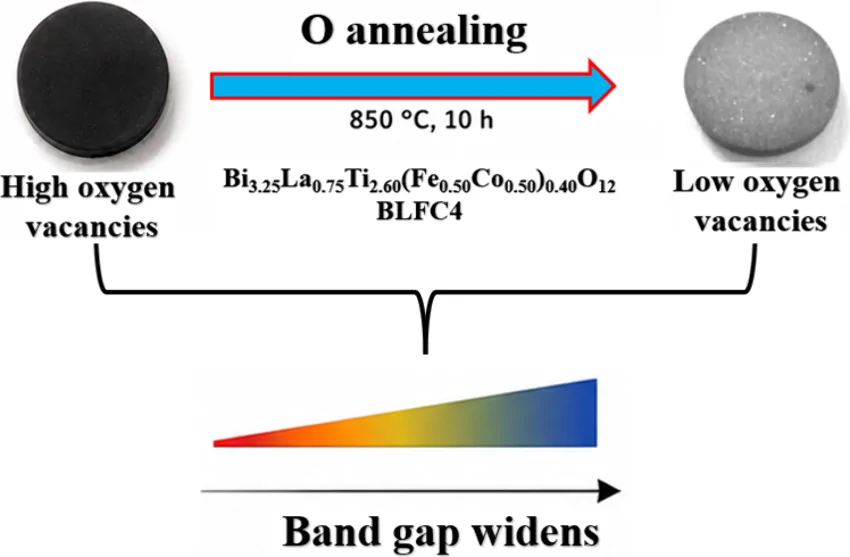

## 1 Introduction

Research on ferroelectric and multiferroic oxides has attracted considerable attention because their intrinsic spontaneous polarization can facilitate the separation of photogenerated charge carriers and generate photovoltages exceeding the semiconductor band gap, offering a promising route toward enhanced solar energy conversion.^1–3^ Developing efficient photoferroelectric materials remains challenging, however, because the key requirements for photovoltaic performance, a narrow band gap, large remanent polarization, and low leakage current, are often mutually conflicting: reducing the band gap typically introduces defect-assisted conduction pathways and band-tail states that increase leakage and degrade device performance.^4–7^ Designing ferroelectrics whose electronic structure can be tailored without compromising structural stability or ferroelectric functionality is therefore essential.^1^ Among ferroelectric oxide families, Aurivillius compounds, with the general formula (Bi_2_O_2_)^2+^(A_*m*–1_B_*m*_O_3*m*+1_)^2–^, are particularly attractive owing to their layered structure, high Curie temperatures, thermal robustness, and compositional flexibility.^8,9^ Bi_4_Ti_3_O_12_ (BIT), a prototypical member, comprises three perovskite-like layers separated by fluorite-like (Bi_2_O_2_)^2+^ sheets (Figure 1), and exhibits robust ferroelectricity over a broad temperature range, widely exploited for memory, sensing, and energy-conversion applications.^8–11^ Its relatively wide band gap, however, restricts absorption to the ultraviolet region, limiting its effectiveness in visible-light-driven photovoltaic and photocatalytic systems.^12^

**Figure 1:**
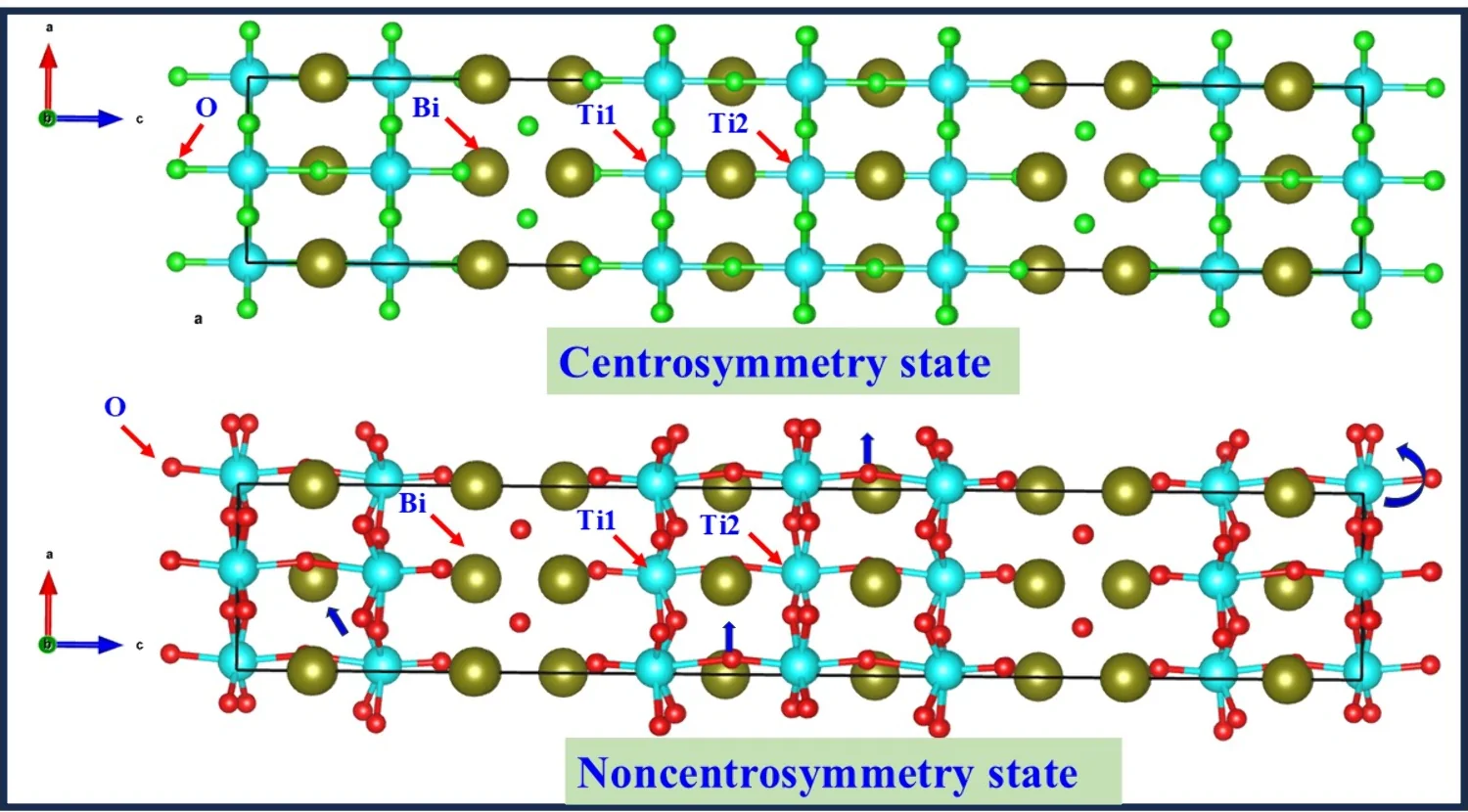
Schematic diagram illustrating the crystal structure of Bi_4_Ti_3_O_12_ Aurivillius compounds in their paraelectric (above the Curie temperature) and ferroelectric (below the Curie temperature) states.

To mitigate this limitation, substitution at both the A and B crystallographic sites has been widely explored. Rare-earth substitution at the A site, particularly La^3+^ and Nd^3+^, increases remanent polarization, fatigue resistance, and charge retention, making La- and Nd-doped BIT (BLT and BNdT) important lead-free ferroelectrics for non-volatile memories.^13,14^ These A-site modifications, however, induce only modest electronic changes: Even Bi_3.25_La_0.75_Ti_3_O_12_ retains an optical band gap exceeding ∼3.0 eV, too high for efficient solar harvesting.^15^ This limited impact has motivated B-site transition metal engineering instead, where Fe and Co incorporation effectively narrows the band gap from ∼2.75 eV in undoped BIT to ∼1.8–2.0 eV while preserving the layered framework.^16–19^ Related defect- and dopant-engineering strategies for tuning electronic and optical properties have also proven effective in other materials classes, including dopant-tunable band gaps in double perovskites established through combined experimental and computational approaches^20^ and defect-engineered oxide heterostructures for photodetection and photocatalysis,^21^ underscoring defect engineering as a general design principle across material systems.

This band-gap narrowing, however, generally comes at a cost: we recently showed that heavy Co doping in Nd-modified bismuth titanate (BNdT–Co, 0–30%) reduces the band gap from 3.22 to 1.88 eV but collapses the remanent polarization from 10.26 to 0.74 μC/cm^2^, via a rise in oxygen vacancy concentration from 2.27 to 15.16%.^22^ Notably, this trade-off proved largely reversible upon oxygen annealing, which simultaneously widened the gap back to 2.6 eV, raised the polarization to 8 μC/cm^2^, and lowered the vacancy fraction to ∼10%^22^ indicating that oxygen vacancy population, rather than cation doping itself, is the dominant control parameter. What remains unclear is the microscopic origin of this effect at the electronic structure level, and whether it depends on which transition-metal site a given vacancy occupies. These questions were inaccessible to our earlier, purely experimental study of BNdT–Co, which established the reversibility of vacancy-driven band-gap narrowing but could not resolve its electronic structure origin or site dependence. Beyond band-gap narrowing, transition-metal substitution introduces localized 3d states near the band edges that influence dielectric, ferroelectric, and transport properties without destroying the underlying Aurivillius structure.^18,19,23^ The perovskite blocks also exhibit pronounced, defect-sensitive octahedral distortions,^24^ and the concentration and distribution of oxygen vacancies together with the associated Ti, Fe, and Co oxidation-state changes can substantially alter the electronic density of states and govern the resulting functional performance.^18,19,23,24^

In La/Fe/Co-modified BIT (BLFC), La primarily stabilizes the Aurivillius lattice while Fe and Co act as the principal electronic modifiers through their partially occupied 3d orbitals. First-principles studies indicate that defect-free BLFC remains semiconducting, with band-gap reduction arising mainly from transition-metal-derived states rather than a true metallic transition. The mechanism behind the much larger band-gap narrowing observed experimentally and its reversal upon oxygen annealing, as we observed directly in BNdT–Co^22^ remains poorly understood, since most prior work has attributed the optical response predominantly to cation substitution with little attention to oxygen vacancy defects. In particular, it is unclear whether band-gap reduction is driven by Fe/Co co-doping itself or by accompanying vacancy-induced defect states, how vacancy concentration, local distortion, valence states, and band-edge reconstruction are interrelated, and whether vacancies near Fe- versus Co-centered octahedra behave differently. To address these questions, the present work combines X-ray diffraction, Raman spectroscopy, X-ray photoelectron spectroscopy, optical absorption, and density functional theory on defect-free and oxygen-deficient Fe/Co co-doped, La-modified BIT (BLFC4) ceramics. Its novelty lies in establishing a direct mechanistic link between oxygen vacancy chemistry, structural evolution, and band-edge reconstruction extending, at the microscopic level, the purely experimental defect-recovery behavior we previously reported in BNdT–Co.^22^ We show that the optical band gap in BLFC4 is governed predominantly by oxygen vacancy concentration rather than cation co-doping alone, and reveal a distinct site-dependent defect behavior: vacancies adjacent to Fe-centered octahedra preserve semiconducting character, whereas those near Co-centered octahedra generate states near the Fermi level and drive the system toward a nearly metallic regime. These findings provide new microscopic insight into band-gap evolution in BLFC and identify oxygen vacancy engineering as a critical design strategy for Aurivillius photoferroelectrics, photovoltaics, and optoelectronics.

## 2 Experimental Section

Polycrystalline Bi_3.25_La_0.75_Ti_2.60_(Fe_0.50_,Co_0.50_)_0.40_O_12_ (BLFC4) ceramics were prepared by a conventional solid-state reaction route. Bi_2_O_3_(99.97%), TiO_2_ (99.8%), La_2_O_3_ (99.99%), Co_3_O_4_(99.8%) and Fe_2_O_3_ (99.8%) powders (Alfa Aesar, U.S.A.) were used as starting materials. The oxides were thoroughly mixed and ground in acetone using an agate mortar and pestle, then dried at 100 °C for 1 h. The dried mixture was calcined in air at 850 °C for 8 h with heating and cooling rates of 5 °C min^–1^. The calcined powders were reground for 1 h and pressed into pellets of ∼10 mm diameter and ∼1 mm thickness using 1 wt % poly­(vinyl alcohol) as a binder. The green pellets were heated to 500 °C at 2 °C min^–1^, held for 1 h to remove the binder, and then sintered in air at 950 °C for 4 h with heating and cooling rates of 5 °C min^–1^. The sintered samples were subsequently annealed in flowing oxygen at 850 °C for 0, 2, 4, 6, 8, and 10 h. Phase purity and crystal structure of the annealed and non-annealed specimens were examined by powder X-ray diffraction (Bruker D8), and the diffraction data were refined using the FullProf suite; crystal structures were visualized using VESTA (version 3.5.2). Micro-Raman spectra were recorded in the 50–950 cm^–1^ range on a Horiba Jobin-Yvon iHR550 spectrometer equipped with a CCD detector and a 514 nm Ar^+^ laser. X-ray photoelectron spectroscopy (XPS) was performed on a Scienta Omicron system with a monochromatic Al Kα source (*h*ν = 1486.7 eV, 280 W) in fixed analyser transmission (FAT/CAE) mode, operated at a pass energy of 30 eV, with the analyser at normal emission relative to the sample surface, and the binding-energy scale was calibrated against the adventitious C 1s peak at 284.8 eV, to determine the valence states of the constituent elements. Optical characterization was carried out using a Shimadzu UV-2600 UV–vis spectrophotometer over the 200–1200 nm wavelength range for all investigated samples.

## 3 First-Principles Calculations

The ab initio calculations were performed within density functional theory (DFT) using the Vienna *Ab initio* Simulation Package (VASP).^25–27^ The interaction between valence and core electrons was described by the projector augmented-wave (PAW) method.^28,29^ The following valence configurations were treated explicitly: Bi (6s^2^6p^3^), Ti (3d^3^4s^1^), O (2s^2^2p^4^), Fe (3d^7^4s^1^), Co (3d^8^4s^1^), and La (5s^2^5p^6^5d^1^6s^2^). Exchange–correlation effects were treated within the generalized gradient approximation (GGA) using the PBEsol functional,^31^ which was chosen because previous comparative first-principles work on Bi_5_FeTi_3_O_15_ has shown that PBEsol yields a more reliable structural description for Aurivillius phases than standard PBE.^30,32^ A plane-wave kinetic-energy cut off of 550 eV was used in all calculations. Brillouin-zone integrations were carried out with Γ-centered 4 × 4 × 1 *k*-point meshes for structural relaxations and 12 × 12 × 1 meshes for electronic structure calculations. All structures were fully relaxed, including both lattice parameters and internal atomic positions, until the residual forces were smaller than 2 × 10^–3^ eV Å^–1^ and the total-energy convergence threshold reached 2 × 10^–8^ eV. In some cases, the self-consistent cycle initially converged to metastable configurations; to avoid such undesired local minima, small random displacements of about 0.25 Å were applied to the unrelaxed structures prior to optimization. Owing to the low symmetry of the doped configurations, full relaxation leads to small deviations from the ideal orthorhombic cell, particularly in the interaxial angles, but these deviations remain smaller than 1% and do not alter the overall orthorhombic character.

Strong correlation effects associated with the Fe 3d and Co 3d states were treated using the Dudarev GGA + *U* formalism,^33^ in which a single effective Hubbard parameter accounts for the on-site Coulomb interaction. In general, *U* values are chosen on a semi-empirical basis, guided by experimental observables such as the band gap, but inappropriate values can introduce spurious electronic features.^34^ In this work, the purpose of the first-principles calculations is to elucidate the microscopic mechanism governing the evolution of the electronic structure induced by oxygen vacancies introduced through co-doping by La, Fe, and Co, rather than to reproduce the absolute experimental optical band gap. In particular, the calculations are intended to identify the relative changes in the electronic structure associated with different defect and doping configurations. Therefore, the *U* values used for the Fe and Co 3d states were not selected by fitting the calculated band gap to the experimental optical gap. Instead, they were calibrated by benchmarking the GGA + *U* electronic structure against hybrid-functional (HSE06) calculations performed for the simpler Fe and Co single-doped systems. Although HSE06 generally provides a more accurate description of the electronic structure of transition-metal oxides, their computational cost becomes prohibitive for the large supercells required to describe the co-coped configurations. Consequently, HSE06 calculations were employed only as a benchmark for the simpler systems, whereas the calibrated GGA + *U* approach was adopted for the complete atomistic models investigated in this work. This comparison between GGA + *U* and HSE06 indicated that *U* = 3.5 eV for Fe and *U* = 3.0 eV for Co provide the closest agreement with the HSE06 electronic structure while maintaining a computationally efficient framework for the co-doped structures. A complete set of results obtained for different *U* values and computational details are presented in the Supplementary Information. To represent dopant disorder in the Aurivillius lattice, the lowest energy Fe–Co co-doped configuration obtained from the site preference analysis was taken as the reference structure. Then, La atoms were distributed over the Bi sublattice using the Special Quasirandom Structures (SQS) method.^35^ This approach was initially created to model substitutional disorder in finite supercells.^35^ and later extended via the stochastic optimization scheme of van de Walle and co-workers,^36^ constructs chemically disordered configurations by matching the most relevant correlation functions of an ideal random alloy. The SQS generation workflow was implemented using the ICET framework.^37^ Starting from the Bi_4_Ti_3_O_12_ parent compound,^38^ the target co-doped composition was modelled as Bi_3.25_La_0.75_Ti_2.60_Fe_0.20_Co_0.20_O_12_, corresponding to La substitution at the Bi site and the replacement of Ti by an equimolar Fe/Co mixture. Full structural parameters for the SQS configurations are given in the Supplementary Information.

## 4 Results and Discussion

### 4.1 XRD Analysis

Figure 2 shows the room-temperature XRD patterns of BLFC4 ceramics annealed in oxygen for 0–10 h. All patterns can be indexed to the Aurivillius Bi_4_Ti_3_O_12_-type structure (JCPDS 35-0795) with orthorhombic symmetry (space group *B*2*cb*),^9,10,38,39^ and no extra reflections are detected at any annealing time. This confirms that Fe/Co co-doping and subsequent oxygen annealing preserve a single-phase Aurivillius lattice, in agreement with related Co/Fe-doped Aurivillius compounds reported elsewhere.^40^ Structural refinement using FullProf (Figure 3), based on the Rietveld method,^41^ yields an excellent match between observed and calculated profiles, with low *R* factors and χ^2^ values (Table 1), indicating that the chosen structural model describes the data reliably, consistent with the refinement approach used for related Co/Fe co-doped Aurivillius ceramics.^42^ The obtained X-ray diffraction refined profiles of the annealed (BLFC4) ceramics with different durations are shown in Figure 3. All refined X-ray diffraction patterns show intense Bragg peaks and no extra reflections, confirming that all BLFC4 samples remain single phase after oxygen annealing. The refined lattice parameters change only slightly but in a systematic way with oxidation time (Table 1 and Figure 4): *a* parameter is almost unchanged, whereas *b* increases from 5.4367 Å (0 h) to 5.4470 Å (10 h) and *c* from 32.8201 to 32.8233 Å. As a result, the unit-cell volume expands from 965.11 to 967.28 Å^3^ and the *b*/*a* ratio rises from 1.00516 to 1.00680, a trend consistent with the systematic lattice-parameter evolution reported across the Aurivillius homologous series with compositional and defect changes.^43^ This evolution is consistent with the gradual shift of the (117) reflection toward lower 2θ values in Figure 2b and indicates a slight lattice expansion and enhanced in-plane anisotropy as the oxidation time increases. A plausible origin of this behaviour is the change in oxygen vacancy concentration during annealing at 850 °C in an oxygen atmosphere.^18–23^ Oxygen diffusion into the BLFC4 lattice gradually fills pre-existing vacancies, which increases the average lattice parameters and relaxes internal strain. The reduction of defect-induced strain also contributes to the sharpening of the diffraction peaks at longer annealing times. Thus, extended oxygen annealing simultaneously expands and relaxes the lattice, providing the structural evidence for the defect healing that is confirmed by XPS and supported by the DFT analysis of vacancy effects.

**Figure 2:**
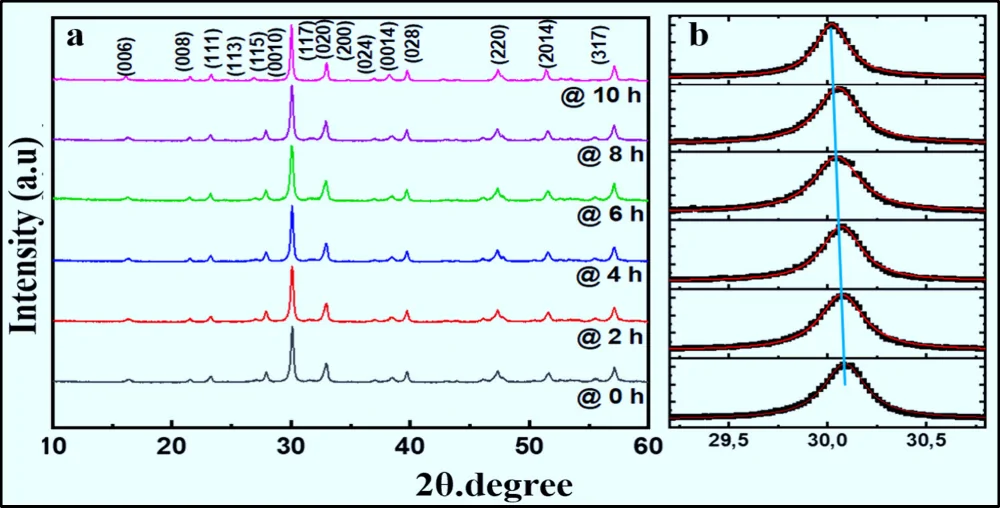
(a) Room-temperature XRD patterns of annealed BLFC4 ceramic and (b) shifting and splitting of the (117) peak with increased annealing time from 0 to 10 h.

**Figure 3:**
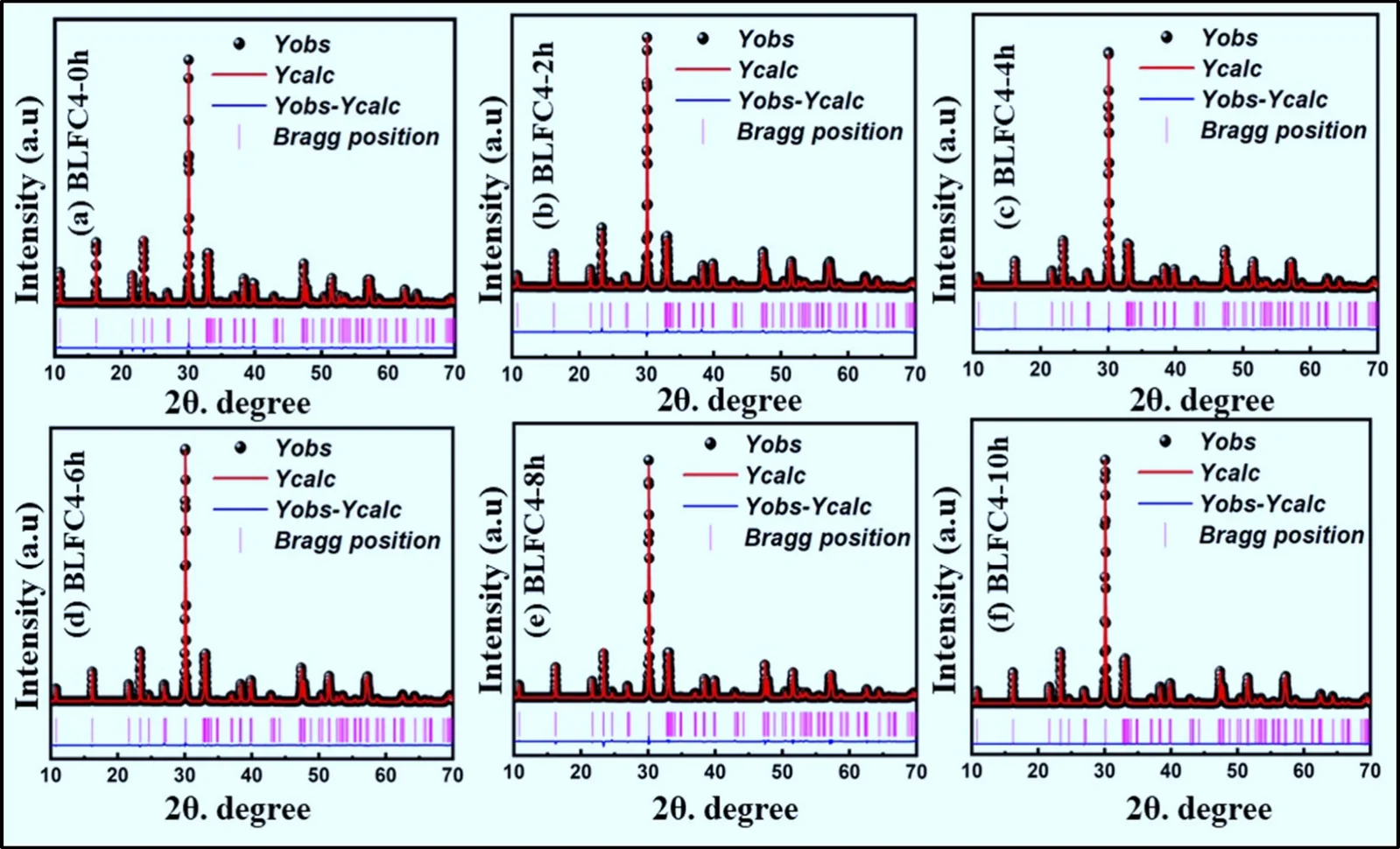
X-ray dffraction (XRD) aalysis of BLFC4 ceramics annealed under an oxygen atmosphere for varying durations. Panels a–f illustrate the subsequent information. The solid black circles signify the measured intensity; the red line denotes the calculated intensity; the blue curve depicts the disparity between the experimental and computed intensities; and the pink vertical bars indicate the Bragg positions.

**Table 1:** Rietveld Refinement Results of X-ray Diffraction Data of Bi_3.25_La_0.75_Ti_2.60_(Fe_0.50_,Co_0.50_)_0.40_O_12_ Annealed in Oxygen Atmosphere at Different Times and Measured at Room Temperature

sample	BLFC4-0h	BLFC4-2h	BLFC4-4h	BLFC4-6h	BLFC4-8h	BLFC4-10h
crystal system	orthorhombic	orthorhombic	orthorhombic	orthorhombic	orthorhombic	orthorhombic
*a* (Å)	5.4088	5.4093	5.4097	5.4093	5.4095	5.4102
*b* (Å)	5.4367	5.4376	5.4379	5.4432	5.4464	5.447
*c* (Å)	32.8201	32.8212	32.8216	32.8227	32.8231	32.8233
*V* (Å^3^)	965.1086	965.39	965.5264	966.4284	967.0441	967.2816
*b*/*a*	1.00516	1.00523	1.00521	1.00627	1.00682	1.0068
space group	*B*2*cb*	*B*2*cb*	*B*2*cb*	*B*2*cb*	*B*2*cb*	*B*2*cb*
SG number	41	41	41	41	41	41
density (g/cm^3^)	7.801	7.811	7.820	7.831	7.871	7.921
*R*_p_	6.82	3.61	7.12	3.37	3.71	3.71
*R*_wp_	8.66	6.12	7.81	6.26	9.23	9.10
*R*_ex_	8.22	4.27	7.11	4.87	7.53	7.42
χ^2^	1.1099	2.0542	1.2065	1.6523	1.5024	1.5040

**Figure 4:**
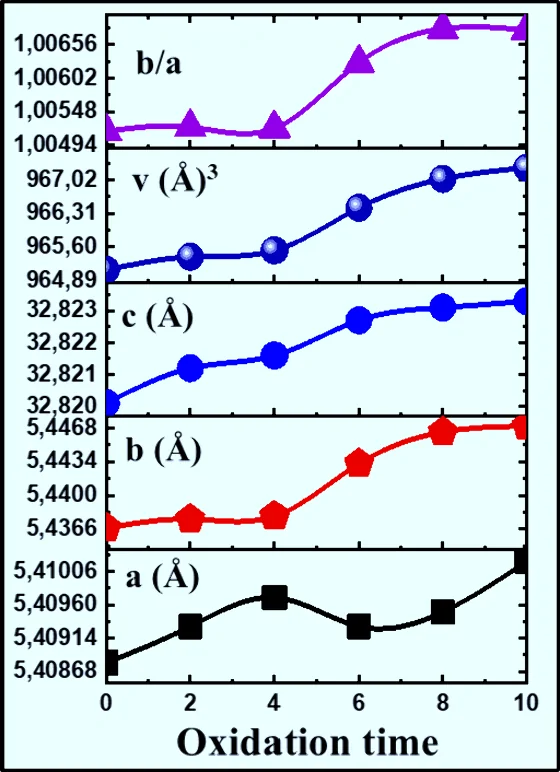
Variation of lattice constant (*a*, *b*, and *c*), unit cell volume (*V*), and orthorhombicity (*b*/*c*) with annealing time in the oxygen atmosphere for the BLFC4 ceramics.

The degree of octahedral distortion was quantified using the bond length distortion index^19^

$$\Delta d=\left(\frac{1}{6}\right)\underset{n=1,6}{\sum }\left[{\left(\frac{d_{n}-\mathrm{d̅}}{d}\right)}^{2}\right]$$

where *d*_*n*_ is the individual metal–oxygen bond lengths within a given (inner or outer) TiO_6_ octahedron, *d̅* is the mean bond length of that octahedron, and *n* is the coordination number (*n* = 6). Δ*d*_in_ and Δ*d*_out_ are evaluated separately for the inner Ti(2)-centered) and outer Ti(1)-centered octahedra, respectively, using the bond lengths listed in Table 2.

**Table 2:** Some Critical Bond Lengths, Bond Angles, and Inner and Outer Octahedral Distortion of the Annealed BLFC4 Ceramics with Different Annealing Times

sample	BLFC4-0h	BLFC4-2h	BLFC4-4h	BLFC4-6h	BLFC4-8h	BLFC4-10h
[Ti(1)–O1] × 4 (Å)	1.91371(8)	1.91449(8)	1.91561(8)	1.91678(8)	1.91758(8)	1.92032(8)
[Ti(1)–O3] × 2 (Å)	2.00181(11)	2.00251(11)	2.00206(11)	2.00255(11)	2.00159(11)	2.0035(11)
[Ti(2)–O3] (Å)	2.23519(13)	2.23564(13)	2.23508(13)	2.23563(13)	2.23556(13)	2.23669(13)
[Ti(2)–O4] (Å)	2.16153(12)	2.1638(12)	2.16288(12)	2.16341(12)	2.16237(12)	2.16443(12)
[Ti(2)–O5] × 4 (Å)	2.02693(8)	2.02789(8)	2.02791(8)	2.02879(8)	2.02925(8)	2.03063(8)
Ti(2)–O(5)–Ti(2) (deg)	156.037(3)	158.014(3)	162.032(3)	165.039(3)	167.067(3)	172.056(3)
tilting angle, ω (deg)	18.9815(3)	18.493(3)	15.985(3)	12.4805(3)	6.4665(3)	3.972(3)
Δ*d*_in_ × 10^–4^	1.68753	1.68319	1.62313	1.59634	1.53163	1.49795
Δ*d*_out_ × 10^–4^	8.10832	8.0842	8.03418	8.00168	7.94525	7.92709
*E*_g_ (eV)	1.35	1.57	1.89	2.03	2.15	2.20

More detailed information on the local environment of the perovskite-like slabs is provided by the bond lengths and distortion parameters in Table 2.^9,10^ The outer Ti(1)–O(1) × 4 distance increases from 1.9137(8) Å (0 h) to 1.9203(8) Å (10 h), and Ti(1)–O(3) × 2 evolves from 2.0018(11) to 2.0035(11) Å. On the inner octahedra, Ti(2)–O(3), Ti(2)–O(4), and Ti(2)–O(5) also exhibit small but systematic increases. These changes indicate a slight elongation and relaxation of both inner and outer TiO_6_ octahedra with annealing, rather than any abrupt structural transition. The connectivity of the inner octahedra changes more strongly. The Ti(2)–O(5)–Ti(2) bond angle increases from 156.03° in BLFC4-0h to 172.06° in BLFC4-10h, while the octahedral tilting angle ω decreases from 18.98° to 3.97°. In parallel, the inner and outer octahedral distortion factors Δ*d*_in_ and Δ*d*_out_ decrease from 1.6875 × 10^–4^ and 8.1083 × 10^–4^ to 1.4979 × 10^–4^ and 7.9271 × 10^–4^, respectively. Together, these trends show that oxygen annealing straightens the Ti–O–Ti linkages and reduces both tilt and distortion of the TiO_6_ network, driving the perovskite-like blocks toward a much more regular octahedral arrangement while maintaining the global orthorhombic symmetry, consistent with the coupling between octahedral distortion and oxygen vacancy concentration reported for related Co/Fe co-doped Aurivillius ferroelectrics.^19–24^

This structural evolution fits naturally with the defect chemistry and the DFT results. Experimentally, oxygen annealing reduces the concentration of oxygen vacancies and converts Ti^3+^/Fe^2+^/Co^2+^ into Ti^4+^/Fe^3+^/Co^3+^, as shown by XPS. In the DFT calculations, vacancy-free BLFC configurations exhibit only weak octahedral distortions, whereas oxygen vacancies especially when located next to Co-centered octahedra produce pronounced local distortions, consistent with Jahn–Teller-active behavior expected for octahedrally coordinated Co^2+^/Co^3+^ centers,^44^ and introduce Co-derived states near the Fermi level. The observed increase in Ti(2)–O(5)–Ti(2) angle and decrease in Δ*d*_in_/Δ*d*_out_ with oxidation time therefore indicate that oxygen annealing is progressively removing the most disruptive vacancy-related defects, relaxing the octahedral framework, and preparing the structural conditions under which the band gap can widen, as confirmed by the optical and DFT analyses.

### 4.2 Raman Analysis

Figure 5 shows the Raman spectra of BLFC4 ceramics annealed in oxygen for 0–10 h and their Gaussian deconvolution into seven modes (P1–P7); the corresponding peak positions and FWHM values are listed in Tables 3 and 4. As in orthorhombic Bi_4_Ti_3_O_12_-based Aurivillius phases, the 50–950 cm^–1^ range contains several overlapping modes derived from the tetragonal representation (6A_1g_ + 2B_1g_ + 8E_g_), with E_g_ modes splitting into B_2g_/B_3g_ components due to the orthorhombic distortion.^11,45,46^ All fitted modes exhibit systematic changes in position and/or FWHM with annealing time, indicating progressive defect annealing rather than random spectral fluctuations.

**Figure 5:**
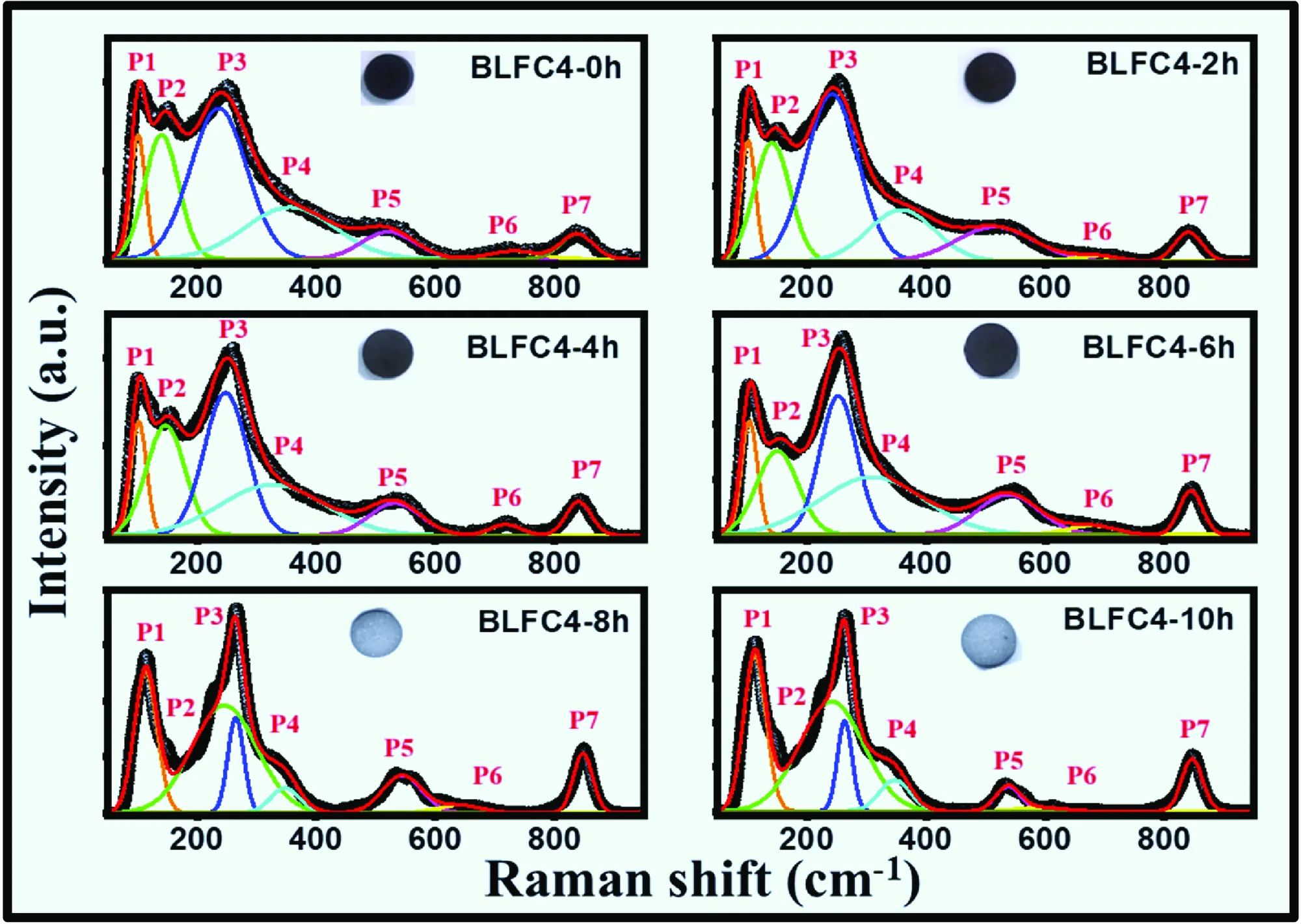
Raman signal fitting curves for annealed BLFC4 ceramics using Gaussian curves. The experimental data are shown by the thick black curve; the Gauss fitting is shown by the colored curves; and the overall intensities of the Gauss lines are shown by the solid red curve. Insets show photographs of the corresponding pellets.

**Table 3:** Raman Peak Positions (cm^–1^) Obtained from Gaussian Deconvolution for BLFC4 Ceramics Annealed in Oxygen for Different Times

	peak 1	peak 2	peak 3	peak 4	peak 5	peak 6	peak 7
BLFC4-0h	101.07227 ± 0.09041	140.64293 ± 0.041118	236.16016 ± 0.073403	355.83863 ± 0.01968	520.68663 ± 0.128335	731.38076 ± 0.16783	837.41144 ± 0.19075
BLFC4-2h	101.42528 ± 0.09349	141.73925 ± 0.04622	242.05244 ± 0.06298	358.46045 ± 0.041755	515.23135 ± 0.13121	684.43978 ± 0.17486	839.77773 ± 0.12754
BLFC4-4h	102.17474 ± 0.011379	147.40973 ± 0.048827	248.0093 ± 0.029782	326.75938 ± 0.078519	532.17896 ± 0.18395	718.06936 ± 0.12651	840.25211 ± 0.18444
BLFC4-6h	103.80118 ± 0.010946	150.5779 ± 0.079708	252.8749 ± 0.028359	309.77322 ± 0.053628	537.79297 ± 0.17162	677.58658 ± 0.11171	844.19654 ± 0.17278
BLFC4-8h	113.91819 ± 0.013375	245.80221 ± 0.0748	265.08537 ± 0.020352	347.84747 ± 0.059036	543.9201 ± 0.12648	640.8587 ± 0.17984	847.61113 ± 0.25956
BLFC4-10h	112.80638 ± 0.014846	241.39843 ± 0.021014	263.53224 ± 0.023491	347.43326 ± 0.071057	537.55778 ± 0.14203	594.06082 ± 0.165047	846.41992 ± 0.18322

**Table 4:** Raman Peaks Full Width Half Maximum Induced from Gauss Fitting for BLFC4 Ceramics Annealed in the Oxygen Atmosphere for Different Times

	peak 1	peak 2	peak 3	peak 4	peak 5	peak 6	peak 7
BLFC4-0h	24.64918 ± 0.12939	57.07045 ± 0.05247	94.57582 ± 0.010156	150.48072 ± 0.099315	89.96911 ± 0.0214	81.0917 ± 0.091652	53.90551 ± 0.04349
BLFC4-2h	24.78337 ± 0.1406	60.47518 ± 0.07309	88.20641 ± 0.050079	111.53691 ± 0.075909	130.46205 ± 0.06659	75.45588 ± 0.065594	45.59192 ± 0.08055
BLFC4-4h	26.31198 ± 0.19205	64.74999 ± 0.04881	72.09243 ± 0.028524	174.14798 ± 0.070084	86.39849 ± 0.04765	51.43285 ± 0.073466	42.48959 ± 0.08965
BLFC4-6h	26.71837 ± 0.1946	69.28431 ± 0.026001	61.55585 ± 0.01365	175.80364 ± 0.022636	103.5715 ± 0.01042	79.27174 ± 0.030121	38.36667 ± 0.05912
BLFC4-8h	38.22178 ± 0.18063	109.63359 ± 0.061033	26.80123 ± 0.053715	47.92122 ± 0.020491	65.48582 ± 0.099727	70.98644 ± 0.066427	32.10119 ± 0.02885
BLFC4-10h	37.47788 ± 0.1146	108.1133 ± 0.089348	24.4885 ± 0.062554	51.56155 ± 0.01765	41.4199 ± 0.074563	71.19287 ± 0.087122	34.72879 ± 0.00531

The low-frequency modes P1 and P2 (≈100–150 cm^–1^), primarily associated with Bi^3+^ vibrations and A site lattice motions,^47,48^ both blue shift with annealing. P1 moves from 101.07 cm^–1^ (0 h) to 112.81 cm^–1^ (10 h) (+11.74 cm^–1^, +11.6%), while its FWHM increases from 24.65 to 37.48 cm^–1^ (+52%). Despite being the largest relative broadening, P1 remains a well-resolved shoulder in Figure 5, consistent with a wider distribution of Bi–O environments as oxygen are incorporated rather than loss of short-range order. P2 shifts from 140.64 to 150.58 cm^–1^ between 0 and 6 h (+9.94 cm^–1^) and broadens from 57.07 to 69.28 cm^–1^, but at 8–10 h the fitting assigns much higher apparent positions and FWHM (≈245/≈110 cm^–1^), which most likely reflect increased overlap with the intensifying P3 envelope rather than a new mode. The P1/P2 blue shift and P1 broadening thus support a stiffening and increased heterogeneity of Bi related vibrations in an oxygen rich lattice, consistent with the modest expansion of the unit cell and the increase of b/a revealed by XRD, while the P2 is best treated as a deconvolution artifact. The intermediate frequency modes P3–P5 (230–550 cm^–1^), assigned to Ti/(Fe,Co)–O bending and stretching vibrations,^14,49^ show clear signatures of lattice relaxation. P3, a torsional Ti/(Fe,Co)–O bending mode derived from E_g_ → B_2g_/B_3g_, shifts from 236.16 to 263.53 cm^–1^ (+27.37 cm^–1^, +11.6%) while its FWHM narrows from 94.58 to 24.49 cm^–1^ (−74.1%). This near monotonic blue shift and strong line width collapse indicate reduced disorder and phonon scattering in the B site framework, and are directly visible as the P3 hump evolves into a sharp peak in Figure 5. P4 and P5 behave similarly but less perfectly: P4 stabilizes near 347–348 cm^–1^ at 8–10 h after earlier fluctuations, with its FWHM falling from ≈175 cm^–1^ (6 h) to ≈50 cm^–1^ (8–10 h), whereas P5 shows a modest net blue shift (+16.87 cm^–1^) and a FWHM reduction from the as sintered value to 41.42 cm^–1^ at 10 h. The pronounced narrowing of P4/P5 after 6–8 h correlates with XRD evidence for a Ti(2)–O(5)–Ti(2) bond angle increase (156° → 172°) and octahedral tilt reduction (≈19° → 4°), implying that most of the TiO_6_ network relaxation occurs in this annealing window. The high frequency modes P6 and P7 (>650 cm^–1^) originate from Ti/(Fe,Co)­O_6_ stretching vibrations.^50,51^ Initially, P6 and P7 are broad and relatively weak (731.38/837.41 cm^–1^; FWHM ≈ 81/54 cm^–1^). With annealing, P6 undergoes a large overall red shift to 594.06 cm^–1^ at 10 h (−137.32 cm^–1^, −18.8%), accompanied by modest, non-monotonic FWHM changes (ending ≈12% narrower than at 0 h). P7, by contrast, shifts only slightly (837.41 → 846.42 cm^–1^, +1.1%) but narrows steadily from 53.91 to 34.73 cm^–1^ (−35.6%), becoming a well-defined peak near 840–847 cm^–1^ in Figure 5. The strong red shift and intensity loss of P6, together with the sharpening and stabilization of P7, suggest that oxygen annealing suppresses or restructures the vacancy distorted, more covalent stretching environment responsible for P6 (likely involving under coordinated B site cations), while the regular Ti/(Fe,Co)­O_6_ stretching represented by P7 becomes dominant. This interpretation is consistent with the decrease in octahedral distortion parameters (Δ*d*_in_ and Δ*d*_out_) and the approach of the Ti(2)–O(5)–Ti(2) linkage toward a nearly linear geometry. Considered together, the B site related modes exhibit a coherent defect annealing pattern: P3, P4, P5, and P7 narrow by 35–74%, while P3 and P6 show the largest absolute position shifts (+27.4 and −137.3 cm^–1^). These trends are characteristic of reduced disorder and diminished phonon scattering from oxygen vacancy induced strain fields, in agreement with similar B-site substitution-driven Raman narrowing reported for related Fe/Co co-doped Aurivillius systems,^52^ and are strongly supported by XPS, which shows a substantial decrease in non-lattice O 1s (22.11% → 4.12%) and in Ti^3+^, Fe^2+^, and Co^2+^ fractions as oxygen annealing progresses. DFT calculations further corroborate this picture: vacancy-free BLFC displays only minor octahedral distortions, whereas Co-adjacent vacancies produce strong local distortions and Co-derived states near the Fermi level, narrowing the band gap toward a nearly metallic regime, while Fe-adjacent vacancies leave the system semiconducting. DFT also predicts stabilization of a high-spin Co^2+^-like state near Co-adjacent vacancies, in agreement with the experimentally observed drop in Co^2+^ content after annealing. The concurrent reduction of octahedral tilt, the 74% narrowing of P3 and 35.6% narrowing of P7, and the widening of the optical band gap (1.35 → 2.20 eV), together with the color change from black/dark gray to light gray, all support the conclusion that oxygen annealing efficiently suppresses Co-adjacent vacancy configurations and drives the lattice toward a more ordered Aurivillius structure. Overall, the Raman data show that oxygen annealing is an effective, quantifiable defect-engineering route in BLFC4 ceramics. Across the seven modes, FWHM values decrease by 12–74% (except for the P1/P2 Bi-related doublet, which broadens by ≈52%) and peak positions shift by up to +27.4 cm^–1^ (P3) or −137.3 cm^–1^ (P6), with most changes occurring between 6 and 10 h. Together with XRD, XPS, and DFT, these results establish that oxygen vacancy concentration and distribution especially around Co-centered octahedra strongly govern both local crystal structure and electronic band-edge properties in BLFC4.

### 4.3 XPS Analysis

The XPS survey spectrum shown in Figure 6 confirms the presence of Bi, La, Ti, Fe, Co, and O on the surface of the BLFC4 ceramics, together with a small C 1s signal used for charge calibration. Because of the limited inelastic mean free path of photoelectrons at these kinetic energies, the XPS-derived compositions represent only the near-surface region and may not be quantitatively representative of the bulk stoichiometry. No angle-resolved XPS or Ar^+^-sputter depth profiling was performed in this study; therefore, the existence of a surface-to-bulk stoichiometry gradient cannot be excluded. Nevertheless, the annealing-induced changes in the Ti^3+^, Fe^2+^, and Co^2+^ concentrations observed by XPS are consistent with the bulk structural evolution revealed by XRD. Specifically, these changes correlate with the reduced octahedral tilt and distortion, as well as the straightening of the Ti(2)–O(5)–Ti(2) bond linkage across the annealing series (Table 2). This agreement suggests, although it does not conclusively prove, that the near-surface defect chemistry broadly reflects the bulk behavior. To establish this relationship quantitatively, future studies should employ depth-resolved XPS or complementary bulk-sensitive techniques such as X-ray absorption spectroscopy (XAS) or electron energy-loss spectroscopy (EELS). This limitation should therefore be considered when interpreting the present XPS results. High resolution spectra for Bi 4f and La 3d (Figure 7a and b) show that the A site cations remain fully oxidized and essentially unaffected by oxygen annealing. The Bi 4f peaks appear at 157.16/162.47, 157.13/162.43, and 157.43/162.74 eV for BLFC4-0h, BLFC4-4h, and BLFC4-10h, respectively, characteristic of Bi^3+^ in Aurivillius oxides.^53,54^ La 3d spectra in Figure 7b exhibit the typical doublet–satellite structure of La^3+^, with La 3d_5_/_2_ main peaks at ∼832.70, 832.85, and 832.88 eV and La 3d_3_/_2_ at ∼849.68, 849.89, and 850.05 eV, accompanied by satellites ∼4 eV higher in binding energy. The small shifts with annealing are within experimental uncertainty and indicate that La maintain a stable 3+ valence independent of oxygen content.^55,56^ The corresponding spin–orbit splitting (5.07, 5.73, and 5.65 eV) confirms the Ti^4+^ assignment.^57^ An additional component at ∼454.6 eV is attributed to Ti^3+^ 2p_3_/_2_.^58^ Quantitative analysis of Ti^3+^ (Table 5) was estimated using the formula reported in the references,^59,60^ and the results show that the Ti^3+^ fraction decreases from 42.68% (0 h) to 31.72% (4 h) and 10.47% (10 h), while Ti^4+^ increases from 57.32 to 68.28 and 86.53%. Thus, oxygen annealing significantly reduces Ti^3+^ type surface defects, which is consistent with the observed change in sample colour from black to grey. This increase in Ti^4+^ content with annealing time is expected, since it reflects the corresponding decrease in oxygen vacancy content in the surface layer upon annealing in the oxygen atmosphere. In contrast, the B site cations and oxygen show pronounced changes with annealing. The Ti 2p spectra (Figure 8a) can be decomposed into Ti^4+^ 2p_3_/_2_ and 2p_1_/_2_ peaks at 456.26/461.33 eV (0 h), 456.09/461.82 eV (4 h), and 456.31/461.96 eV (10 h), in good agreement with TiO_2_.^57^

**Figure 6:**
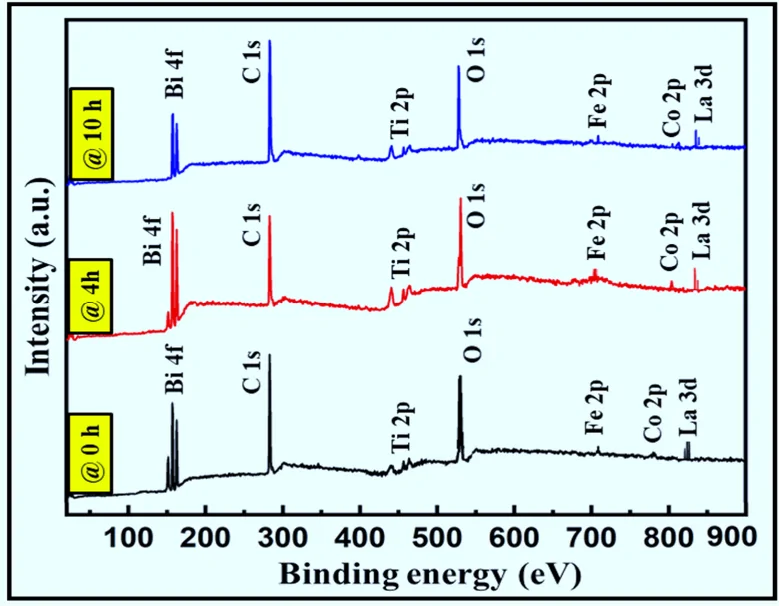
XPS spectra a survey of the BLFC4 ceramics annealed for 0, 4, and 10 h durations.

**Figure 7:**
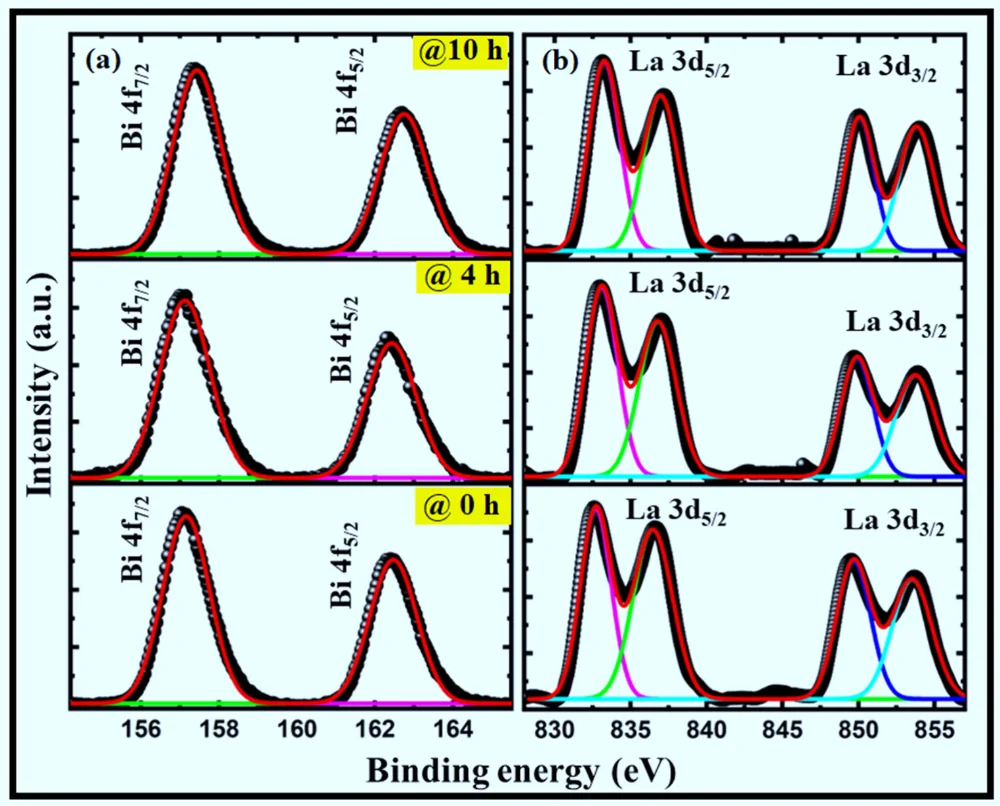
Shows a high-resolution XPS spectrum of (a) Bi 4f and (b) La 3d in BLFC4 ceramics annealed for 0, 4, and 10 h durations.

**Table 5:** XPS-Derived Binding Energies and Relative Concentrations of Lattice Oxygen, Oxygen Vacancies, and Ti/Fe/Co Valence States in BLFC4 Ceramics Annealed in Oxygen for Different Times

	BLFC4-0h	BLFC4-4h	BLFC4-10h
Bi 4f_7/2_ (eV)	157.16	157.13	157.43
Bi 4f_5/2_ (eV)	162.47	162.43	162.74
La 3d_5/2_ (eV)	832.70	832.85	832.88
La 3d_5/2_ (sat) (eV)	836.45	836.76	836.96
La 3d_3/2_ (eV)	849.68	849.89	850.05
La 3d_3/2_ (sat) (eV)	853.51	853.71	853.82
Ti^4+^ 2p_3/2_ (eV)	456.26	456.09	456.31
Ti^3+^ 2p_3/2_ (eV)	454.60	454.55	454.83
Ti^4+^ 2p_1/2_ (eV)	461.33	461.82	461.96
Ti^4+^ content (%)	57.32	68.28	86.53
Ti^3+^ content (%)	42.68	31.72	10.47
O_L_ 1s (lattice)	529.80	529.84	529.83
O_V_ 1s (non-lattice) (eV)	531.15	531.17	531.34
O_L_ content (%)	77.89	81.4	95.88
O_V_ content (%)	22.11	18.60	4.12
Fe^2+^ 2p_3/2_ (eV)	709.56	709.11	708.99
Fe^3+^ 2p_3/2_(eV)	711.77	711.02	711.06
Fe^3+^ 2p_1/2_ (eV)	724.26	724.14	724.17
Fe^3+^ content (%)	79.90	85.98	93.97
Fe^2+^ content (%)	20.10	14.02	6.030
Co^3+^ 2p_3/2_ (eV)	781.17	779.45	779.01
Co^2+^ 2p_3/2_ (eV)	786.03	783.16	783.45
Co^3+^ 2p_1/2_ (eV)	796.09	795.08	795.03
Co^3+^ content (%)	83.90	85.00	97.50
Co^2+^ content (%)	16.10	15.00	2.50

**Figure 8:**
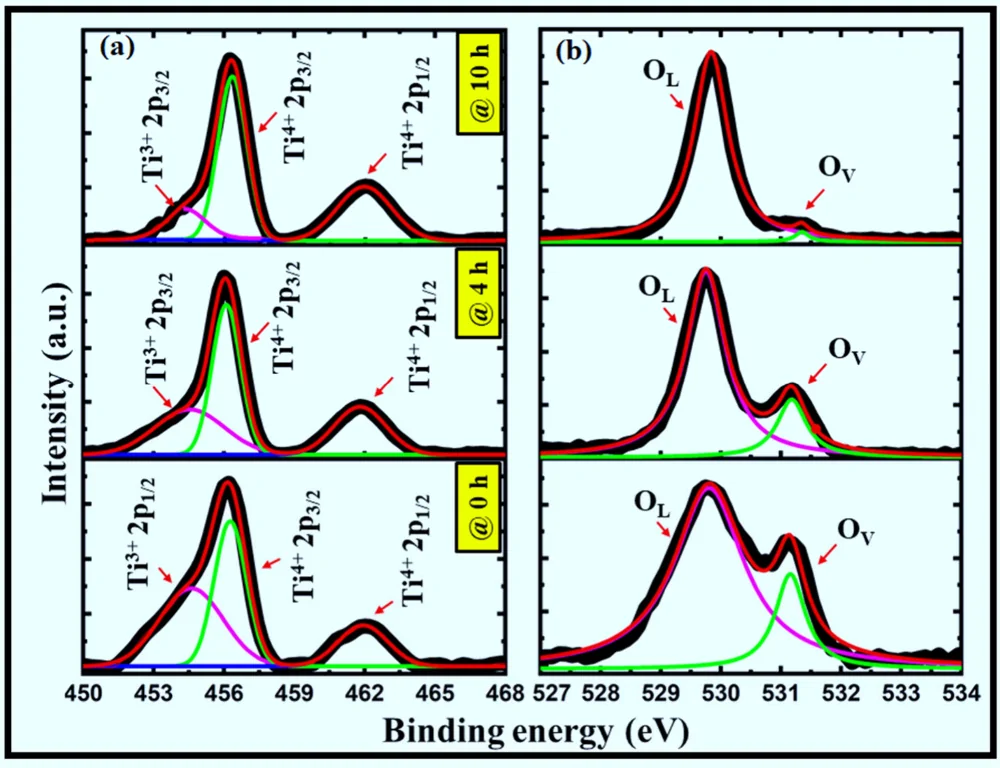
Shows a high-resolution XPS spectrum of (a) Ti 2p and (b) O 1s in BLFC4 ceramics annealed for 0, 4, and 10 h durations.

The O 1s spectra (Figure 8b) further clarify the evolution of oxygen defects. They can be fitted with two components: a low binding energy peak at ∼529.8 eV corresponding to lattice oxygen (O_L_) and a higher binding energy peak at ∼531.1–531.3 eV associated with non-lattice oxygen (O_V_), including oxygen vacancies and adsorbed species.^61,62^ The relative area of O_V_ decreases strongly with annealing time, from 22.11% for BLFC4-0h to 18.60% at 4 h and only 4.12% at 10 h, while O_L_ increases from 77.89 to 95.88% (Table 5). This evolution demonstrates that oxygen annealing at 850 °C effectively fills oxygen vacancies and stabilizes the oxygen sublattice in a near stoichiometric configuration. According to this observation, oxidation of Fe^3+^ or Co^3+^ in a charge-neutral system will result in readjustments in atomic positions. The results further emphasize the importance of oxygen vacancies and Ti^3+^ defects in modifying band-gap energy, as discussed in the following sections. An increase in oxygen vacancies during air sintering may change Fe^3+^ and Co^3+^ valence states. This occurs if the liberated electrons are bound to Fe^3+^ and Co^3+^ in the system. Following a charge change, Fe^3+^ and Co^3+^ will become Fe^2+^ and Co^2+^, as illustrated below.^63–66^

$${\mathrm{Fe}}^{3+}+e^{\prime }↔{\mathrm{Fe}}^{2+}$$

$${\mathrm{Co}}^{3+}+e^{\prime }↔{\mathrm{Co}}^{2+}$$

A high-resolution XPS spectrum of Fe 3p and Co 2p for the samples that were annealed for 0, 4, and 10 h, respectively, was performed to validate the valence state of Fe and Co cations in the BLFC4 crystal, as shown in Figure 9a and b. Figure 9a exhibits the core level spectra of Fe 2p in BLFC4 sample before and after annealing in oxygen at different times.

**Figure 9:**
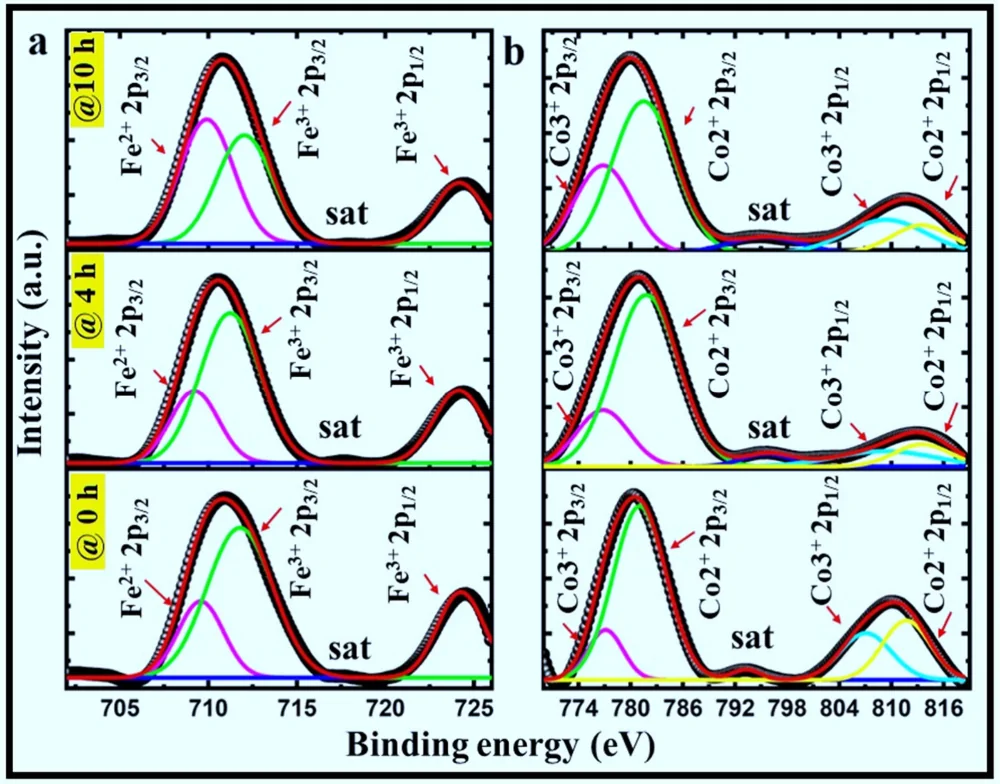
High-resolution XPS spectrum of (a) Fe 2p and (b) Co 2p in BLFC4 ceramics annealed for 0, 4, and 10 h durations.

The XPS spectra of Fe reveal the presence of 2p_3/2_ and 2p_1/2_ doublets, which originate from the spin-orbit coupling.^67,68^ These results are consistent with the valence-state behavior reported for related Co/Fe co-substituted Aurivillius multiferroic ceramics.^69^ The XPS spectra of Fe^2+^ 2p_3/2_ are seen at ∼709.56, 709.11, and 708.99 eV for samples annealed at 0, 4, and 10 h, respectively. However, the XPS spectra of Fe^3+^ 2p_3/2_ and Fe^3+^ 2p_1/2_ are observed at 711.77, 711.02, and 711.06 eV and 724.26, 724.14, and 724.17 eV for samples annealed at 0, 4, and 10 h, respectively. Although the peak positions do not change significantly in different annealed samples, the area under the curve shows appreciable change. The calculated concentration ratio of Fe^3+^/Fe^2+^ in air and oxygen-annealed samples was 79.90:20.10, 85.98:14.02, and 93.97:6.03 for samples annealed at 0, 4, and 10 h, respectively. Considering the various possible experimental errors, the calculated total error in the estimated values was ±1%. The XPS spectra of Co 2p in the Figure 9b reveal the presence of Co^3+^ 2p_3/2_ and Co^3+^ 2p_1/2_ at 781.17, 779.45, and 779.01 eV and 786.03, 783.16, and 783.45 eV for the samples before oxygen annealed and after oxygen annealed for 4 and 10 h, respectively. While the XPS spectra of Co^2+^ 2p_3/2_ were observed at 786.03, 783.16, and 783.45 eV for the samples annealed in oxygen for 0, 4, and 10 h, respectively. From fitting results of the Co 2p XPS spectra, the concentration ratio of Co^3+^/Co^2+^ was 83.9:16.1, 85.00:15.00, and 97.50:2.50 for the sample annealed in oxygen for 0, 4, and 10 h, respectively. As can be discerned, the BLFC4 sample without annealing in oxygen has higher Fe^2+^ (20.10%) and Co^2+^ (16.10%) content than that annealed in oxygen, consistent with the broader, red-shifted B-site Raman modes (P6 and P7) observed in the as-sintered sample and their progressive blue shift and narrowing as these reduced-valence species are oxidized upon annealing. Generally, co-dopants Fe and Co cations enter into the BLaT lattice to substitute B-site Ti^4+^ cations in the form of Fe^2+^, Fe^3+^, and Co^2+^ or Co^3+^ states because the radii of Fe^2+^, Fe^3+^, and Co^2+^ or Co^3+^ are close to that of Ti^4+^ (0.605 Å).^70^ It has been evidenced that Fe and Co ions can stabilize in multiple valence states, such as 2+ and 3+ in a BLaT-based system, and their relative ratio depends on annealing time.

Furthermore, the coexistence of Co^2+^ and Co^3+^ cations in BLaT crystal will affect the crystal lattice due to the difference in valence and radius. Fe and Co ions substituting for Ti^4+^ will generate oxygen vacancies in the lattice, shrinking the unit cell.^71,72^ Besides, the incorporation of smaller radius Fe^3+^ and Co^3+^ cations in the B site are also helpful for reducing unit cell volume, as we observed in our recent articles.^59^ In this sense, the substitution by Fe^3+^ and Co^3+^ in place of Ti red shifts the Ti–O-related Raman modes relative to undoped BIT, consistent with literature reports.^69,73^ Figure 10 summarizes how oxygen annealing modifies the defect chemistry of BLFC4. In Figure 10a, the fraction of lattice oxygen O (black curve) increases almost linearly with annealing time, from 77.89% at 0 h to 81.4% at 4 h and 95.88% at 10 h, while the non-lattice oxygen O associated with oxygen vacancies (red curve) decreases from 22.11 to 18.60 and finally 4.12%. This clear anti-correlation confirms that annealing at 850 °C in oxygen efficiently fills vacancies and restores a nearly stoichiometric oxygen sublattice. Figures 10b–d show the corresponding evolution of the B-site cation valence states. For Ti (Figure 10b), the Ti^4+^ content increases from 57.32% (0 h) to 68.28% (4 h) and 86.53% (10 h), whereas Ti^3+^ decreases from 42.68 to 31.72 and 10.47%. Similar behaviour is observed for Fe (Figure 10c): Fe^3+^ rises from 79.90 to 85.98 and 93.97%, while Fe^2+^ drops from 20.10 to 14.02 and 6.03%. For Co (Figure 10d), the Co^3+^ fraction increases from 83.90 to 85.00 and 97.50%, with a corresponding decrease of Co^2+^ from 16.10 to 15.00 and 2.50%. These opposite trends between 3+ and 2+ states demonstrate that oxygen annealing oxidizes Ti, Fe, and Co toward their higher valence, in direct response to the reduction of oxygen vacancy donors. Taken together, Figure 10 confirms a coherent defect-compensation mechanism: as oxygen is incorporated and O_V_ is eliminated, electrons associated with vacancies and Ti^3+^/Fe^2+^/Co^2+^ are removed, driving the system toward a Ti^4+^/Fe^3+^/Co^3+^-dominated state. This chemical evolution accounts for the lattice expansion and reduced octahedral tilt seen by XRD, the sharpening and blue shift of Ti/(Fe,Co)–O Raman modes, and the widening of the optical band gap. It also matches the DFT results, which show that vacancy-free Fe/Co-doped Aurivillius phases remain semiconducting with modest distortions, whereas oxygen vacancies especially those adjacent to Co stabilize Co^2+^-like high-spin states and Co-derived bands near the Fermi level, pushing the system toward a nearly metallic regime. The experimentally observed decrease of O_V_ and Fe^2+^/Co^2+^ fractions with oxygen annealing therefore indicates that these Co-adjacent vacancy configurations are being removed, directly leading to structural relaxation, improved vibrational coherence and restoration of a wider, more intrinsic band gap.

**Figure 10:**
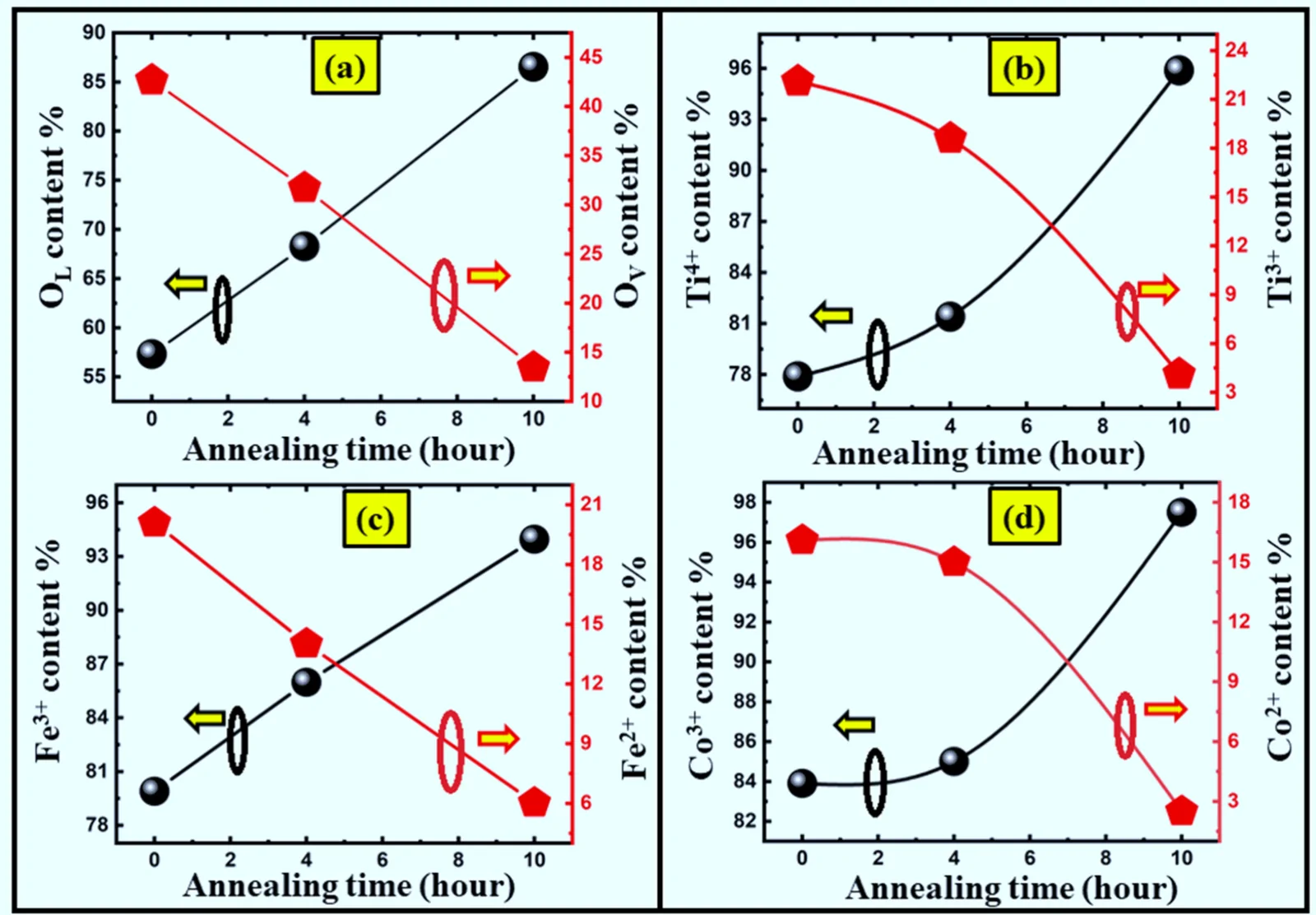
(a) Variation of the oxygen lattice and oxygen vacancies content, (b) variation of the Ti^4+^ and Ti^3+^ content, (c) variation of the Fe^3+^ and Fe^2+^ content, and (d) variation of the Co^3+^ and Co^2+^ content with oxygen annealing times.

### 4.4 Optical Analysis

The optical response of BLFC4 ceramics is highly sensitive to their defect chemistry and octahedral distortions in the Aurivillius type lattice. Oxygen annealing at 850 °C induces a systematic blue shift of the absorption edge (Figure 11a), and Tauc plots (Figure 11b)^74^ show that the optical band gap *E*_g_ increases from approximately 1.35 eV for BLFC4-0h to 1.57 eV (2 h), 1.89 eV (4 h), 2.03 eV (6 h), 2.15 eV (8 h) and 2.20 eV (10 h). Quantitatively, this represents a band gap widening of about 0.85 eV over the oxidation sequence. This continuous increase in *E*_g_ indicates that oxidation progressively removes defect related sub band gap transitions and drives BLFC4 toward its intrinsic semiconducting band structure. In the as sintered BLFC4-0h sample, the layered perovskite slabs contain a high concentration of oxygen vacancies and reduced cations. XPS analysis of the O 1s core level shows that the fraction of non-lattice oxygen associated with vacancies (O_V_) is 22.11%, while lattice oxygen (O_L_) accounts for 77.89%. After annealing for 4 and 10 h, O_V_ decreases to 18.60% and 4.12%, respectively, and O_L_ increases to 81.4 and 95.88%. The Ti 2p spectra reveal that Ti^3+^ contributes 42.68% of the Ti signal at 0 h, decreasing to 31.72% (4 h) and 10.47% (10 h), while Ti^4+^ increases from 57.32 to 68.28 and 86.53%. Similar trends are observed for Fe and Co: Fe^2+^ drops from 20.10 to 6.03% and Fe^3+^ rises from 79.90 to 93.97%, while Co^2+^ decreases from 16.10 to 2.50% and Co^3+^ increases from 83.90 to 97.50%. These quantitative changes confirm that oxygen annealing strongly reduces vacancy concentration and stabilizes Ti, Fe, and Co in their higher oxidation states.

**Figure 11:**
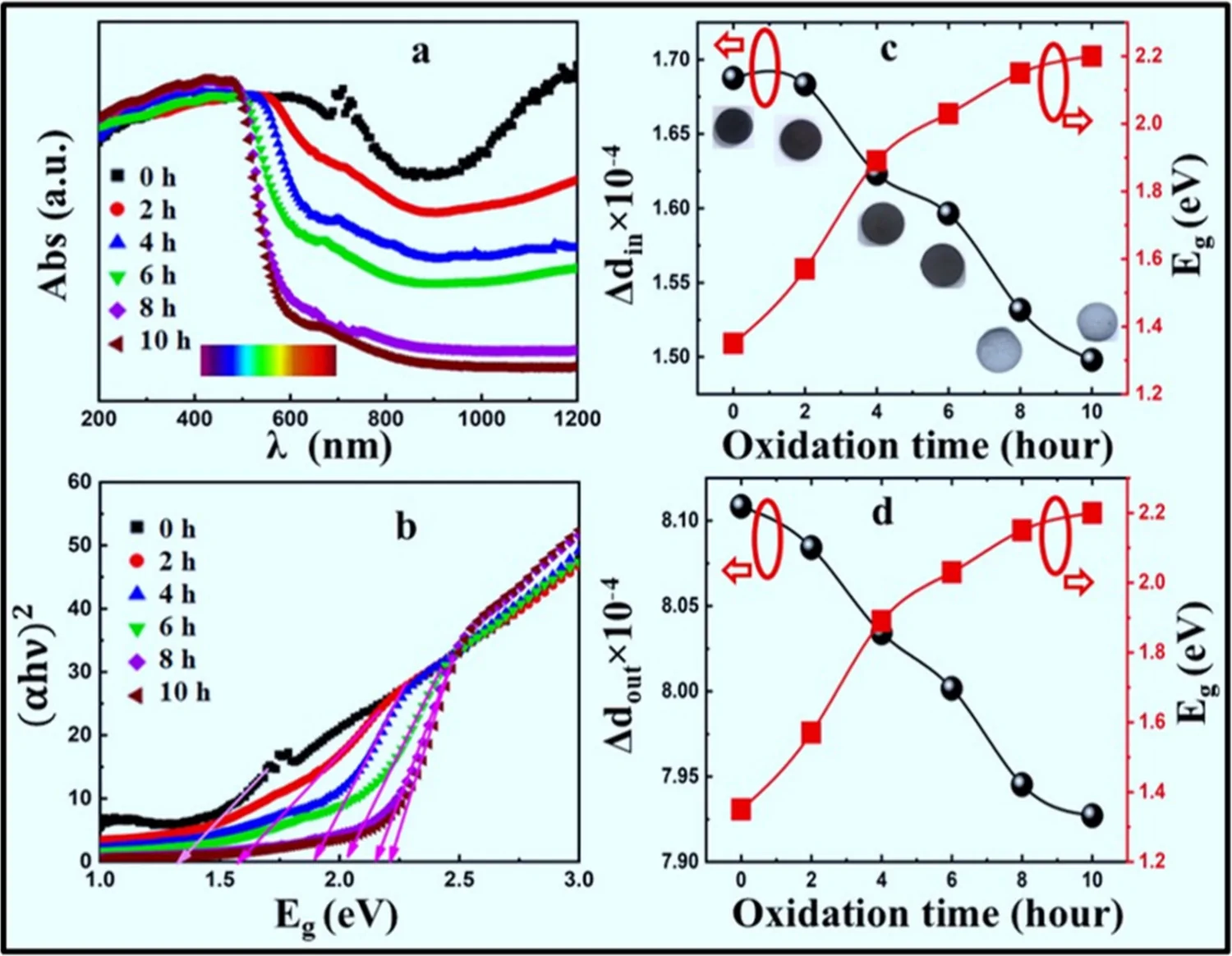
Correlation between optical absorption, octahedral distortion, and oxidation time for BLFC4 ceramics annealed in oxygen for 0–10 h. (a) Room-temperature UV–vis absorption spectra showing the systematic blue shift of the absorption edge with increasing annealing time. (b) Corresponding Tauc plots, (α*h*ν)^2^ versus photon energy, with linear extrapolation to the energy axis used to extract the optical band gap *E*_g_ for each annealing time. (c) Inner octahedral distortion parameter Δ*d*_in_ (black spheres, left axis) and optical band gap *E*_g_ (red squares, right axis) plotted against oxidation time, with photographs of the corresponding pellets showing the progressive color change from black/dark gray to light gray. (d) Outer octahedral distortion parameter Δ*d*_out_ (black spheres, left axis) and *E*_g_ (red squares, right axis) plotted against oxidation time. The inverse correlation between Δ*d*_in_/Δ*d*_out_ and *E*_g_ in panels c and d demonstrates that the suppression of octahedral distortion upon oxygen annealing tracks directly with the widening of the optical band gap.

Using Kröger–Vink defect notation,^75^ the dominant non stoichiometric defect in BLFC4 can be written as a doubly charged oxygen vacancy *V*_O_^··^ formed via

$$O_{O}^{\times }⇌V_{O}^{\cdot \cdot }+\frac{1}{2}O_{2}\left(g\right)+2e^{\prime }$$

where O_O_^×^ denotes lattice oxygen and e′ denotes electrons released into the lattice. These electrons are trapped at B-site cations according to,^75^

$${\mathrm{Ti}}_{\mathrm{Ti}}^{\times }+e^{\prime }⇌{\mathrm{Ti}}_{\mathrm{Ti}}^{\prime },{\mathrm{Fe}}_{\mathrm{Fe}}^{\times }+e^{\prime }⇌{\mathrm{Fe}}_{\mathrm{Fe}}^{\prime },{\mathrm{Co}}_{\mathrm{Co}}^{\times }+e^{\prime }⇌{\mathrm{Co}}_{\mathrm{Co}}^{\prime }$$

where Ti_Ti_^′^, Fe_Fe_^′^, and Co_Co_^′^ represent Ti^3+^, Fe^2+^, and Co^2+^ centers, respectively. In the BLFC4-0h sample, the relatively high fractions of Ti^3+^ (42.68%), Fe^2+^ (20.10%) and Co^2+^ (16.10%) reflect a substantial population of *V*_O_^··^ and associated electrons. These defect complexes generate localized states and band tails inside the nominal band gap, consistent with the octahedral distortion and oxygen-vacancy-induced band-gap narrowing reported for related Co/Fe co-doped Aurivillius ferroelectrics,^19^ enabling optical absorption below the intrinsic edge and resulting in the small effective Eg of 1.35 eV and the dark sample colour. Structural refinement and Raman analysis show that oxidation also significantly relaxes octahedral distortions. The inner distortion parameter Δ*d*_in_ decreases from 1.6875 × 10^–4^ (0 h) to 1.5638 × 10^–4^ (4 h) and 1.4979 × 10^–4^ (10 h) (Figure 11c), while the outer distortion Δ*d*_out_ decreases from 8.1083 × 10^–4^ to 8.0152 × 10^–4^ and 7.9271 × 10^–4^ over the same interval (Figure 11d). The Ti(2)–O(5)–Ti(2) angle increases from 156.03° to 168.21° and 172.06°, and the octahedral tilting angle reduces from 18.98° to 8.42° and 3.97°. Raman peak analysis reveals strong narrowing and blue shifting of the B site related modes (P3–P7), particularly P3 and P7, as annealing time increases. These quantitative structural and vibrational changes indicate that filling *V*_O_^··^ and re oxidizing Ti^3+^/Fe^2+^/Co^2+^ greatly reduce local strain and disorder around the Ti/(Fe,Co)­O_6_ octahedra, leading to a more symmetric lattice with straighter B–O–B linkages.

First-principles DFT calculations support this interpretation.^32–35^ for vacancy free Fe/Co doped BLFC, the structure remains semiconducting with spin resolved band gaps of the order of 0.5–0.7 eV in the calculated density of states, and the TiO_6_, FeO_6_ and CoO_6_ octahedra exhibit only moderate distortions. Fe and Co substitution introduce 3d derived bands near the valence and conduction edges, narrowing the gap relative to undoped Bi_4_Ti_3_O_12_ but preserving a clear separation between valence and conduction bands. When an oxygen vacancy *V*_O_^··^ is placed near Fe (adjacent to Fe_Fe′_), the band gap changes but the system remains semiconducting. In contrast, DFT shows that a vacancy adjacent to Co (near Co_Co′_) produces strong local distortion of the CoO_6_ octahedra and introduces Co derived states very close to the Fermi level, driving the material toward a nearly metallic or heavily band tailored regime. These Co adjacent *V*_O_^··^ defects correspond to the most effective sources of in gap and band edge states, directly reducing the band gap and broadening the absorption edge.

The experimental increase of *E*_g_ from 1.35 to 2.20 eV with oxygen annealing can therefore be understood as the macroscopic consequence of shifting the defect equilibria. Annealing in oxygen pushes the reaction^75^

$$O_{O}^{\times }⇌V_{O}^{\cdot \cdot }+\frac{1}{2}O_{2}\left(g\right)+2e^{\prime }$$

back toward O_O_^×^, consuming *V*_O_^··^and e′ and converting Ti_Ti_^′^, Fe_Fe_^′^, and Co_Co_^′^ quantitatively into Ti_Ti_^×^, Fe_Fe_^×^, and Co_Co_^×^. The reduction of O_V_ content from 22.11 to 4.12% and Co^2+^ from 16.10 to 2.50% indicates that the Co adjacent *V*_O_^··^/Co_Co_^′^ defect complexes identified by DFT are being strongly suppressed. As these complexes are removed, the density of mid gap and band tail states falls, the band edges sharpen, and the optical transitions increasingly reflect the intrinsic vacancy free Fe/Co modified bands. This defect healing is directly responsible for the observed band gap widening and the colour change from dark to light with oxidation time.

The Mott–Davis density-of-states picture provides an additional qualitative framework.^76^ In the highly defective BLFC4-0h sample, structural disorder, oxygen vacancies and dangling bonds generate band tails of localized states extending into the gap, which support low-energy absorption and reduce the apparent *E*_g_. Thermal oxidation passivates these unsaturated bonds and reduces the number of tail states. Together with reduced Δ*d*_in_/Δ*d*_out_ and sharpened Raman modes, this indicates improved atomic-scale regularity and a lower tail-state density. As a result, optical transitions originate from cleaner band edges, and the apparent *E*_g_ expands toward the intrinsic value predicted by vacancy-free DFT calculations.

From a device perspective, this defect-controlled band-gap tuning is critical for BLFC4-based applications. The increase of *E*_g_ to ∼2.2 eV, combined with the reduction of Ti^3+^/Fe^2+^/Co^2+^ and *V*_O_^··^, enhances electrical insulation and reduces leakage current, which is beneficial for ferroelectric capacitors and non-volatile memory elements.^13^ At the same time, retaining a controlled number of Fe/Co-derived states near the band edges (without excessive vacancy-induced tails) can be exploited for photocatalytic or photovoltaic functions.^2,39^ Overall, the quantitative correlation between optical data, Kröger–Vink defect chemistry,^75^ XRD/Raman/XPS results and DFT band-structure calculations shows that precise control of oxygen vacancy concentration and location particularly near Co sites is a powerful strategy for engineering both structural and electronic properties in Fe/Co co-doped La-modified bismuth titanate.

### 4.5 DFT Analysis

To establish a microscopic basis for the structural and electronic trends observed experimentally, density functional theory (DFT) calculations were performed on pristine, singly doped, co-doped, and oxygen-deficient Bi_4_Ti_3_O_12_-based configurations, following the methodology described above. The site-preference analysis (Figure 12) shows that La substitutes preferentially at the A site within the pseudo-perovskite block (P1) rather than the fluorite-like Bi_2_O_2_ layer (P2), consistent with the experimentally reported behavior of La-modified bismuth titanate.,^13,14^ For the B-site dopants, both Fe and Co favor the inner perovskite positions [P2, adjacent to Ti(2)] over the outer sites adjacent to the Bi_2_O_2_ layer (P1), although the preference is markedly stronger for Co (Δ*E* = 0.11 eV/f.u. between P1 and P2) than for Fe (Δ*E* = 0.03 eV/f.u.), indicating weaker B-site selectivity for Fe, in line with first-principles cation site-preference trends reported for related Fe-substituted Aurivillius phases.^32^ This calculated preference for inner-site occupancy provides a direct structural rationale for the experimental observation that the Ti(2)–O(5)–Ti(2) linkage the bond most sensitive to Fe/Co occupancy undergoes the largest angular and tilt changes upon oxygen annealing, since the inner octahedra are precisely where Fe/Co-derived distortions and, ultimately, the oxygen vacancy chemistry are calculated to be concentrated. Among the co-doped Fe–Co arrangements considered, the configuration with the largest Fe–Co separation is energetically most favorable, by 0.06 eV/f.u. over the Fe–O–Co nearest-neighbour arrangement and by 0.97 eV/f.u. over an intermediate arrangement, indicating that Fe and Co dopants tend to avoid direct coordination through a shared oxygen atom.

**Figure 12:**
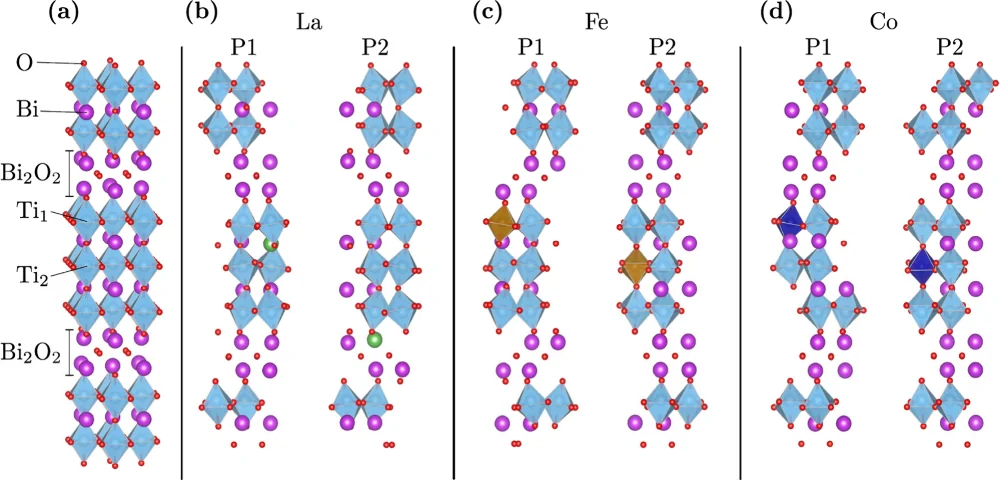
DFT-relaxed structural models used in the site-preference analysis. (a) Experimental reference structure of Bi_4_Ti_3_O_12_. (b) La substitution at the pseudo-perovskite (P1) and fluorite-like (P2) A sites. (c) Fe substitution at the outer (P1) and inner (P2) B sites. (d) Co substitution at the outer (P1) and inner (P2) B sites. Both Fe and Co favor the inner B site (P2), adjacent to the Ti(2) position identified by XRD as the most structurally responsive site upon oxygen annealing.

The electronic structure of the doped systems was analyzed through the projected density of states (PDOS) and depicted in Figure 13a–d. For pristine Bi_4_Ti_3_O_12_, the valence band is dominated by O 2p states with Ti 3d states forming the conduction-band edge, consistent with its semiconducting character, in agreement with previously reported electronic structure calculations of BIT.^17^ Introducing Fe or Co individually narrows the band gap substantially relative to the pristine host: spin-resolved gaps of 1.11/1.56 eV (spin-down/spin-up) are obtained for Fe-doped BIT and 0.81/0.46 eV for Co-doped BIT, with Co producing the stronger reduction in qualitative agreement with the optical gaps reported for Co- and Fe-modified La-doped bismuth titanate (1.85 and 2.02 eV, respectively, versus 2.75 eV for the undoped host).^16–19^ La substitution, by contrast, leaves the gap nearly unchanged (≈2.08 eV) and acts mainly as a structural rather than electronic modifier, consistent with its role as an isovalently A-site dopant.

**Figure 13:**
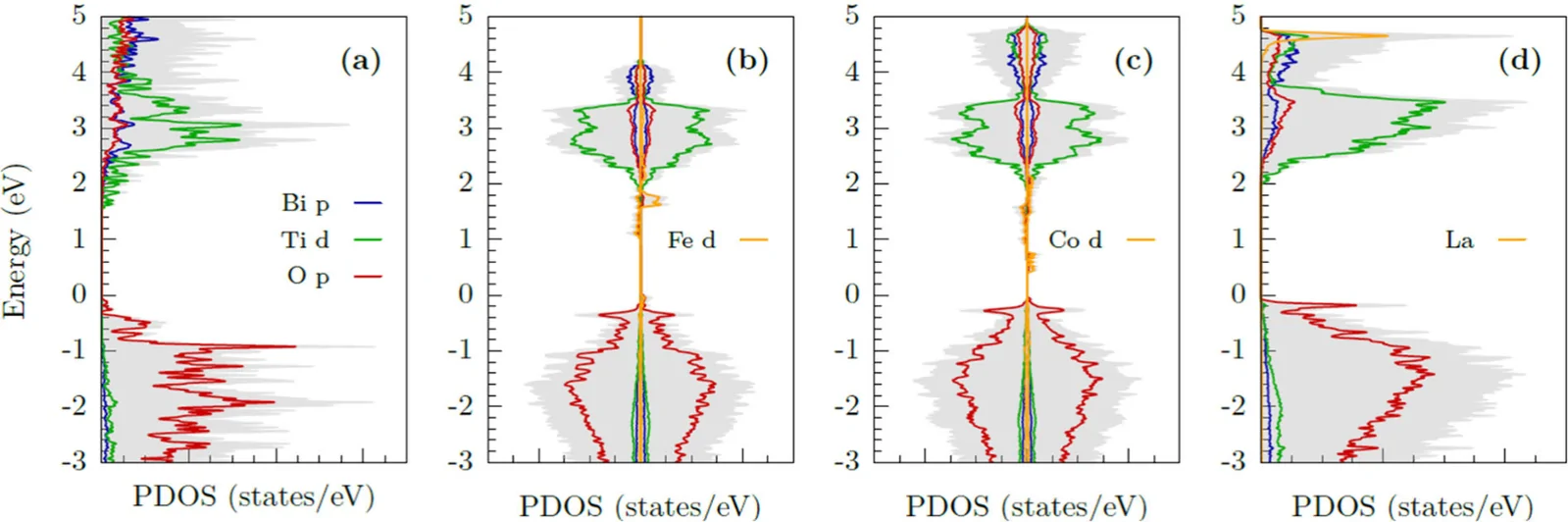
Calculated projected density of states (PDOS) for (a) pristine Bi_4_Ti_3_O_12_ and singly doped configurations with (b) Fe, (c) Co, and (d) La. The shaded region represents the total density of states, while the colored curves indicate the main projected orbital contributions (Bi p, Ti d, O p, and the dopant d/f state). The Fermi energy is set to zero. Fe and Co both introduce dopant-derived states near the band edges that narrow the gap relative to the pristine host, with Co producing the stronger reduction, whereas La leaves the PDOS profile comparatively unchanged.

When Fe and Co are co-doped together in the vacancy-free BLFC structure (Figure 14), their combined effect narrows the gap further, to 0.48 and 0.68 eV for the spin-up and spin-down channels, respectively. We note that these GGA + *U* gaps substantially underestimate the experimental optical gap of the fully annealed sample (2.20 eV), a well-documented shortcoming of GGA (+*U*) functionals; as described in the computational methods, the Hubbard parameters used here were calibrated against HSE06 electronic structure rather than fitted to reproduce the absolute experimental gap, so the calculations should be interpreted in terms of relative trends (site dependence and spin-state evolution) rather than absolute gap magnitude. The orbital-resolved PDOS (Figure 14c and d) shows that the Fe and Co 3d states split into nearly degenerate t_2g_ and e_g_ manifolds,^44^ reflecting the close-to-ideal octahedral coordination of the FeO_6_ and CoO_6_ units in the vacancy-free structure. The calculated local spin moments approximately 2.63 μB for Fe and only 0.005 μB for Co indicate that Fe stabilizes in a high-spin Fe^3+^-like (3d^5^) configuration,^32^ while Co adopts a nearly diamagnetic, low-spin Co^3+^-like (3d^6^) configuration, consistent with the well-established low-spin, diamagnetic ground state of octahedrally coordinated Co^3+^ in oxide systems.^77^ This vacancy-free electronic picture corresponds to the fully oxidized end-member toward which the experimental system evolves upon prolonged oxygen annealing, and it is consistent with the XPS-derived dominance of Fe^3+^ (93.97%) and Co^3+^ (97.50%) after 10 h of treatment.

**Figure 14:**
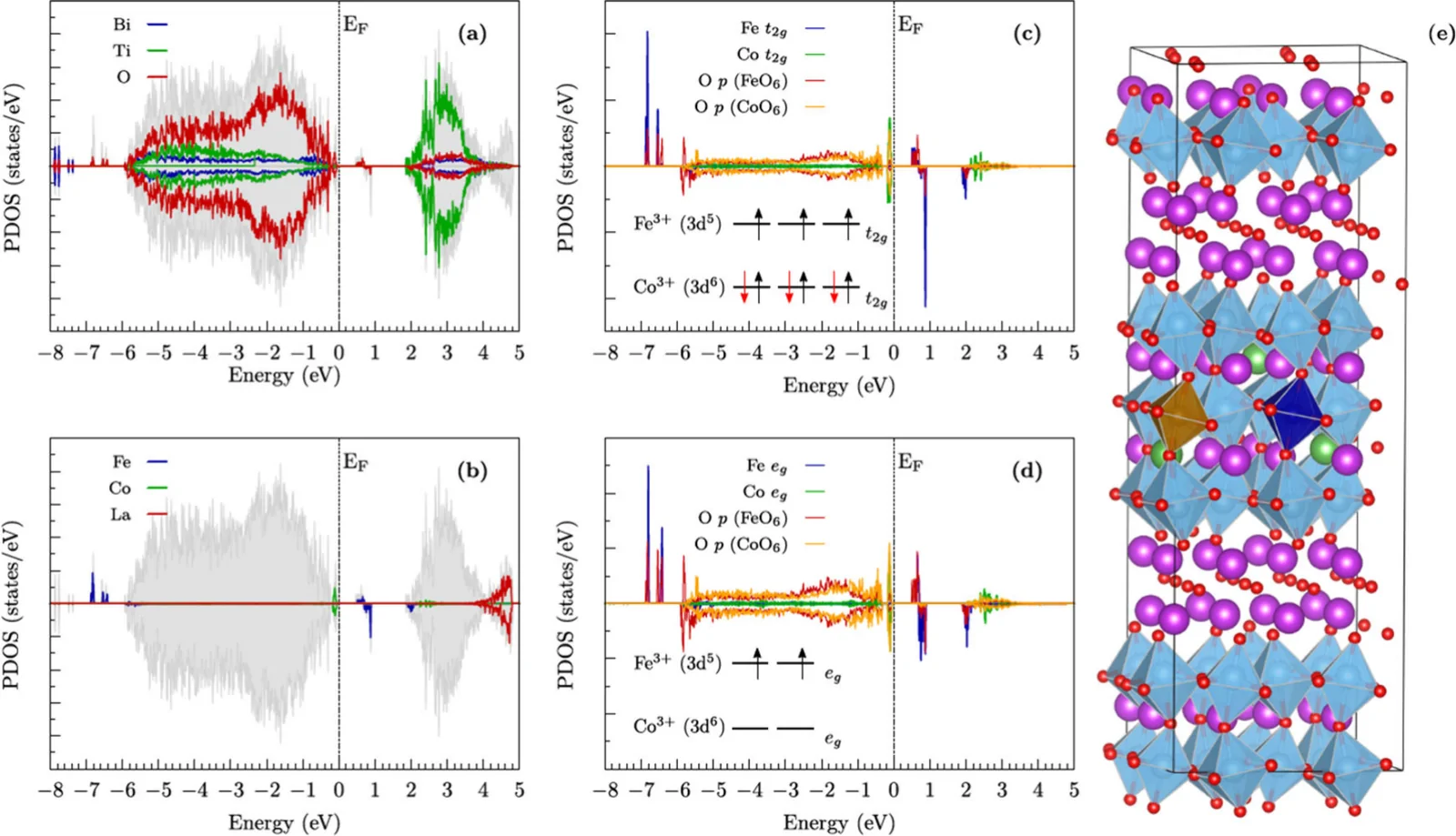
Calculated projected density of states (PDOS) for the fully Fe/Co co-doped, vacancy-free BLFC configuration. (a and b) Atomic contributions from the Bi/Ti/O sublattice and from the Fe/Co/La dopants, respectively. (c and d) Orbital-resolved Fe and Co 3d contributions decomposed into t_2g_ and e_g_ manifolds, with insets showing the high-spin Fe^3+^-like and low-spin Co^3+^-like spin configurations. (e) Relaxed crystal structure of the co-doped BLFC supercell. The Fermi energy is set to zero.

The effect of oxygen vacancies-the central experimental variable in this study-was assessed by introducing a single oxygen vacancy adjacent to either the Fe- or the Co-centered octahedron (Figure 15). The two defect geometries produce markedly different electronic and magnetic responses. A vacancy near Fe (forming a FeO_5_ unit) leaves the local spin configuration essentially unchanged (Co ≈ 0 μB, Fe ≈ +4 μB) and, rather than closing the gap, widens it to 1.38 and 1.56 eV for the spin-up and spin-down channels-wider than the vacancy-free value-because the released electrons are accommodated within a redistributed Fe t_2g_ manifold without generating new states at the Fermi level. A vacancy near Co (forming a CoO_5_ unit), in contrast, produces calculated local spin moments of approximately +2.90 μB for Co and −4.09 μB for Fe, indicating an antiferromagnetic-like alignment and a transition of Co from its low-spin Co^3+^-like state toward a reduced, high-spin Co^2+^-like (3d^7^) configuration.^77^ Critically, this Co-adjacent vacancy introduces Co 3d states directly at the Fermi level, collapsing the gap and driving the system toward a nearly metallic electronic response, consistent with the sensitivity of oxygen-vacancy-induced defect states to the Hubbard *U* treatment reported for related Bi-based oxide systems.^34^ These calculations therefore identify Co-centered octahedra as the dominant site of vacancy-induced electronic disruption in BLFC, while Fe-centered octahedra remain comparatively robust to oxygen loss, in agreement with the experimentally established link between oxygen vacancies, octahedral distortion, and band-gap narrowing in Co/Fe co-doped Aurivillius ferroelectrics.^18,19^ We emphasize that the local spin moments and antiferromagnetic-like Fe–Co coupling reported above are DFT predictions; no experimental magnetometry (SQUID/VSM) was performed in this study, and these magnetic predictions should be regarded as a testable hypothesis rather than a validated result. Direct experimental verification by temperature- and field-dependent magnetization measurements is an important direction for future work.

**Figure 15:**
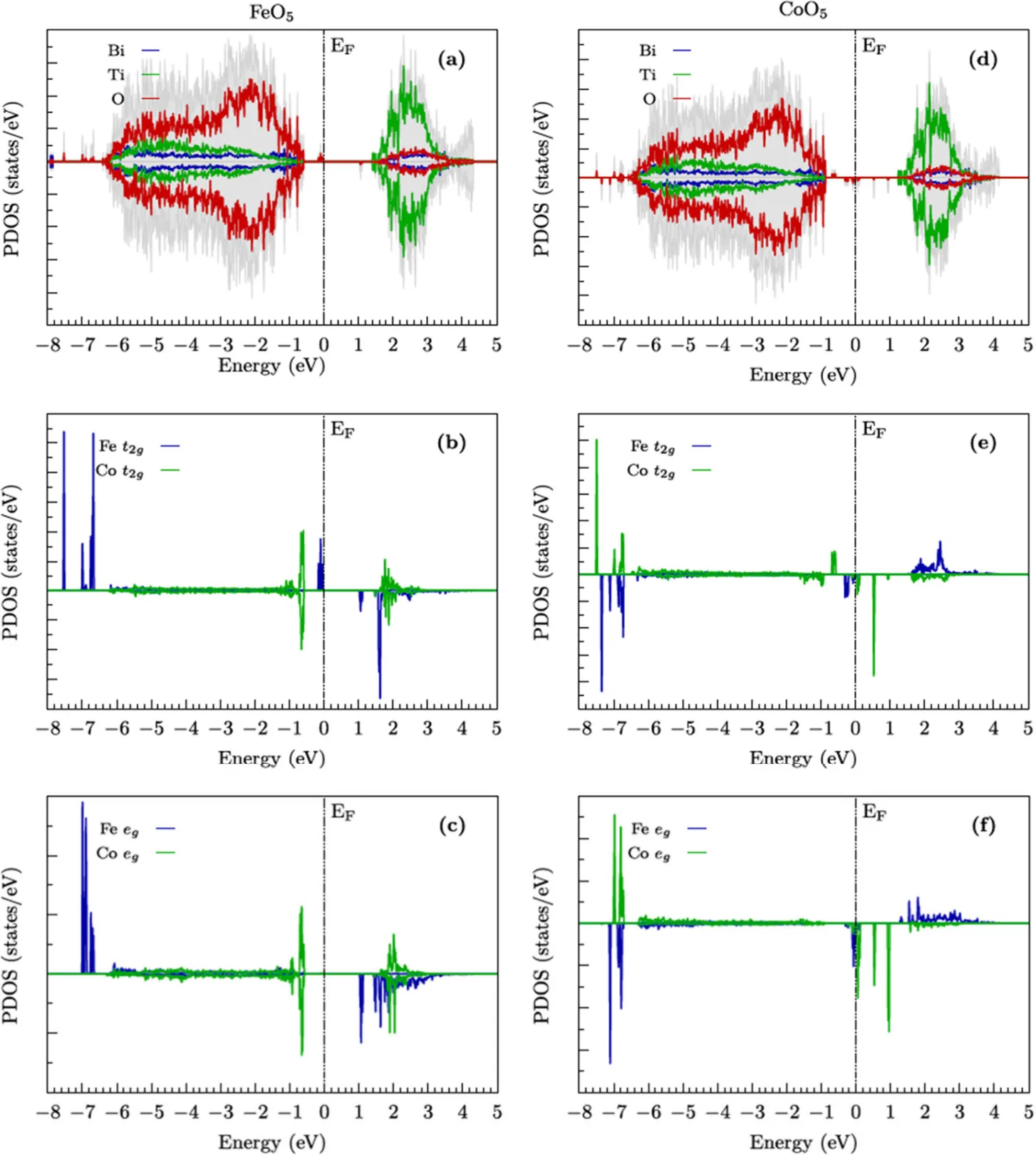
Spin-polarized PDOS calculated for BLFC supercells containing a single oxygen vacancy adjacent to the (a–c) Fe-centered octahedron and (d–f) Co-centered octahedron. Panels a and d show the total and atom-projected DOS; panels b and c and panels e and f show the orbital-resolved t_2g_ and e_g_ contributions for each vacancy configuration. The vacancy near Co introduces Co 3d states at the Fermi level and drives the system toward a nearly metallic response, whereas the vacancy near Fe leaves the system semiconducting. The Fermi energy is set to zero.

This site-dependent DFT picture provides a direct mechanistic explanation for the experimental trends reported above. The XPS-derived reduction of Co^2+^ from 16.10% (0 h) to 2.50% (10 h) markedly larger in relative terms than the corresponding Fe^2+^ reduction is consistent with the calculated preference for vacancy-induced electron trapping at Co rather than Fe sites. The progressive suppression of these Co-adjacent vacancy/Co^2+^ complexes upon oxygen annealing accounts for the pronounced narrowing of the Co-sensitive Raman modes (P3 and P7), the reduction of the octahedral tilt angle from 18.98° to 3.97°, and ultimately the widening of the optical band gap from 1.35 to 2.20 eV, since each of these macroscopic observables tracks the removal of exactly the defect configuration that DFT identifies as most disruptive to the electronic structure. Taken together, the DFT results close the loop between local defect chemistry, octahedral distortion, and band-edge reconstruction, confirming that precise control of oxygen vacancy concentration particularly at Co-centered octahedra is the key microscopic lever for band-gap engineering in Fe/Co co-doped La-modified bismuth titanate, consistent with and extending the structure property relationships previously established for oxygen-vacancy-driven band-gap narrowing in Co/Fe co-doped Aurivillius ferroelectrics.^18,19^

## 5 Conclusion

This work establishes a quantitative, multi-technique picture of how oxygen vacancy chemistry governs band-gap engineering in Fe/Co co-doped, La-modified bismuth titanate (BLFC4). Progressive oxygen annealing (0–10 h, 850 °C) preserves the orthorhombic Aurivillius structure while expanding the unit cell, straightening the Ti(2)–O(5)–Ti(2) linkage, and reducing octahedral distortion (XRD, corroborated by Raman blue-shifting/narrowing of modes P3–P7). XPS shows these tracks a drop in non-lattice oxygen (22.11% → 4.12%) and oxidation of Ti^3+^, Fe^2+^, and Co^2+^, while the optical gap widens from 1.35 to 2.20 eV.

DFT reveals the underlying site-dependent mechanism: a vacancy adjacent to Fe leaves the system semiconducting, whereas a vacancy adjacent to Co introduces Co 3d states at the Fermi level and drives it toward a nearly metallic response. Since Co-adjacent vacancies dominate the in-gap/band-tail states, their removal upon annealing not Fe/Co co-doping itself, is the principal driver of the observed band-gap widening, a conclusion supported consistently across all five techniques. This resolves what our earlier, purely experimental study of BNdT–Co (ref 22) could not: the electronic structure origin of vacancy-driven band-gap tuning, and its dependence on which transition-metal site the vacancy occupies.

Practically, these results identify control of vacancy population at Co-centered sites as a lever for tuning the trade-off between band gap, leakage current, and visible-light absorption. Oxidized BLFC4 (*E*_g_ ≈ 2.2 eV) may offer improved insulation for ferroelectric/non-volatile-memory applications, while the reduced state’s lower energy absorption may suit photocatalytic or photovoltaic use; no device-level electrical, photocatalytic, or photovoltaic measurements were performed here, so both remain plausible directions rather than demonstrated functionalities. Future work combining site-selective vacancy doping with these correlations could refine this design strategy for Fe/Co co-doped Aurivillius oxides.

## Supplementary Material



## Data Availability

All data are confidential and included in the manuscript and its Supporting Information.

## Notes

Additional figures and tables on the site-preference energetics, Fe–Co arrangement configurations, octahedral bond length analysis, and Hubbard *U* parameter selection (band structures and band gaps), as described in the Experimental Section (PDF)
