# Supplementary material for: Oxygen-Vacancy-Controlled Band-Gap Engineering in Fe/Co Co-doped La-Modified Bismuth Titanate: Integrated Experimental and First-Principles Analysis
Source: ACS Omega. 2026 Sep 8;11(37):56450–67. doi: 10.1021/acsomega.6c07692 (PMC13613393; doi:10.1021/acsomega.6c07692)
Supplement: Supplementary file 1 [file ao6c07692_si_001.pdf]

Oxygen-Vacancy-Controlled Band-Gap Engineering in Fe/Co Co-Doped La-Modified Bismuth Titanate: Integrated Experimental and First-Principles Analysis

Mahmoud S. Alkathy[1]*, Danilo P. Kuritza[2], Lais Conservan Nogueira[3], Anibal Thiago Bezerra[1], Person Pereira Neves[1], Vitor F. Barbosa[3], Amatalkareem M. A. Al-Jezbi[4], Flavio Paulo Milton[3], Ricardo Pereira Bonini[3], Rodrigo AR Carvalho, Rafael Alves Lozano[3], Ivair Aparecido dos Santos[5], Alexandre José Gualdi[3], Marcio Daldin Teodoro[3], Fabio L Zabotto[3], Valmor R Mastelaro[6], Manuel H. Lente[7], José A. Eiras[3]*

1. Physics Department, Institute of Exact Sciences- Federal University of Alfenas- MG, 37133-840, Brazil
2. Federal University Paraná - Jandaia do Sul, Jandaia do Sul, 86900-000. Brazil
3. Physics Department, Institute of Exact Sciences-UFSCar- São Carlos, SP, 13565-905, Brazil
4. Center for Environment and Sustainable Energy, Al-Razi University, Sana'a, Yemen
5. Department of Physics, State University of Maringa, Av. Colombo, 5790, Maringa, PR, 87020-900, Brazil
6. Institute of Physics University of São Paulo, São Carlos, CEP 13566-590 São Carlos, SP, Brazil
7. Institute of Science and Technology, Federal University of São Paulo, São José dos Campos, CEP 12231-280, SP, Brazil

Corresponding Authors
mahmoud.kathy@unifal-mg.edu.br
Eiras@df.ufscar.br

Supporting Information

This file contains supplementary computational details, figures, and tables supporting the density functional theory (DFT) analysis presented in the main text, including (i) the relative energetics and lattice parameters obtained in the site-preference analysis, (ii) the structural models and spin states considered for the Fe–Co co-doped supercell arrangements, (iii) the calculated metal–oxygen bond lengths and octahedral distortion parameters, and (iv) the selection of the Hubbard U parameters applied to the Fe-3d and Co-3d orbitals, including comparison with hybrid-functional (HSE06) band structures.

S1. Site-Preference Energetics and Lattice Parameters

Table S1 summarizes the relaxed lattice parameters, unit-cell volumes, b/a ratios, and relative energies (ΔE) obtained for the pristine $Bi_4Ti_3O_{12}$ host and for the singly doped La, Fe, and Co configurations considered in the site-preference analysis (main text, Figure 12). The experimental lattice parameters were taken from the original crystallographic report of Aurivillius[38] and the corresponding Crystallography Open Database entry.[78] Relative energies ΔE are referenced to the lowest-energy configuration within each dopant series.

Configuration	a (Å)	b (Å)	c (Å)	V (Å³)	b/a	ΔE (eV/f.u.)
Experimental	5.4100	5.4480	32.8400	967.92	1.0070	–
Pristine (DFT)	5.3898	5.3895	32.6006	946.98	0.9999	–
La–P1	5.3959	5.4119	32.9472	962.12	1.0030	0.0000

**La–P2**	5.4262	5.3995	32.9865	966.46	0.9951	0.0861
**Fe–P1**	5.4044	5.3961	32.5263	948.54	0.9985	0.0311
**Fe–P2**	5.3767	5.4145	32.6379	950.15	1.0070	0.0000
**Co–P1**	5.3714	5.3889	32.5119	941.08	1.0033	0.1106
**Co–P2**	5.3712	5.3814	32.7534	946.72	1.0019	0.0000

Table S1. Calculated structural parameters and relative energies for pristine and singly doped $Bi_4Ti_3O_{12}$ configurations considered in the site-preference analysis. P1 and P2 denote the outer (fluorite-adjacent / pseudo-perovskite) and inner B-site positions, respectively, as defined in the main text and Figure 12.

S2. Fe–Co Co-Doped Supercell Arrangements

To identify the lowest-energy Fe–Co configuration prior to introducing La via the Special Quasirandom Structures (SQS) approach, three representative Fe–Co arrangements were considered within a 2 × 2 × 1 $Bi_4Ti_3O_{12}$ supercell, both dopants placed at the energetically favoured inner B-sites (Figure S1). In arrangement A1, Fe and Co are nearest neighbours connected through a shared oxygen atom (Fe–O–Co linkage); in A2 and A3, the dopants are progressively more widely spaced.

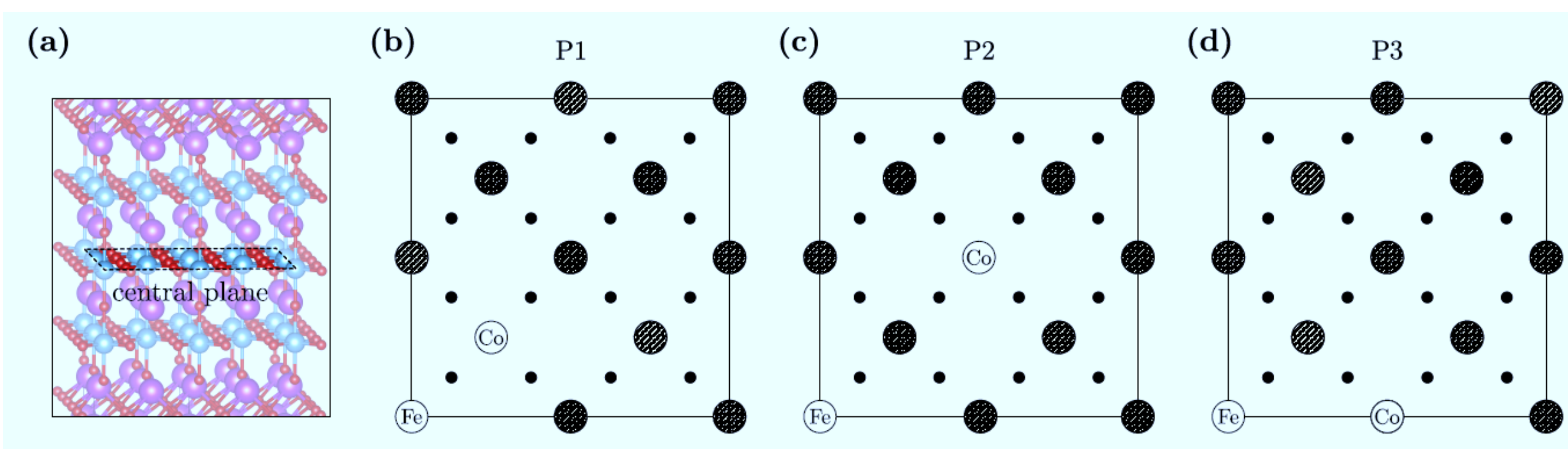

Figure S1. Fe–Co arrangements considered in the co-doped 2 × 2 × 1 $Bi_4Ti_3O_{12}$ supercell. Panel (a) highlights the central perovskite layer where the substitutions were introduced, while panels (b)–(d) show the different Fe/Co arrangements, labelled A1, A2, and A3 according to the relative positions of the dopant atoms. Smaller black circles and larger hatched circles represent O and Ti atoms, respectively.

Table S2 lists the calculated local spin moments (S_z) on the Fe and Co sites, the total spin moment, and the relative energy of each arrangement. Arrangement A3, with the largest Fe–Co separation, is the most stable, by 0.06 eV/f.u. relative to A1 (the Fe–O–Co nearest-neighbour case) and by 0.97 eV/f.u. relative to A2, indicating that Fe and Co dopants energetically favors avoiding direct

coordination through a shared oxygen atom. Fe retains a high-spin-like state in all three arrangements, whereas Co relaxes toward a nearly nonmagnetic, low-spin-like configuration regardless of the initial spin arrangement, consistent with the low-spin Co^{3+} ground state discussed in the main text.

System	S_r(Fe) (μ_B)	S_r(Co) (μ_B)	Total (μ_B)	ΔE (eV/f.u.)
**A1**	2.875	−0.064	2.944	0.0589
**A2**	2.633	−0.001	2.855	0.9727
**A3**	2.616	0.005	2.898	0.0000

Table S2. Calculated local spin moments S_r for the Fe and Co atoms, total spin moment, and relative energy ΔE for the three Fe–Co arrangements shown in Figure S1.

S3. Octahedral Bond Lengths and Distortion Parameters

Table S3 reports the average in-plane (d′) and out-of-plane (d″) metal–oxygen bond lengths, together with their ratio d″/d′, for the Ti-centered and Co/Fe-centered octahedra in the pristine and singly doped $Bi_4Ti_3O_{12}$ structures shown in Figure 12 of the main text. Values close to unity in the d″/d′ ratio indicate a near-regular octahedral environment. As discussed in the main text, the Fe- and Co-centered octahedra remain only weakly distorted relative to an ideal O_h coordination, consistent with the near-degenerate t_{2g} and e_g crystal-field manifolds obtained in the projected density of states (Figure 14) and with the absence of a pronounced Jahn–Teller-type splitting[44] for the vacancy-free, near-ideal octahedral coordination considered here.

System	d′(Ti) (Å)	d″(Ti) (Å)	d″/d′(Ti)	d′(M) (Å)	d″(M) (Å)	d″/d′(M)
**Experimental**	1.919	2.102	1.095	–	–	–
**Pristine (DFT)**	1.935 ± 0.021	1.989 ± 0.164	1.028	–	–	–
**Fe–P1**	1.976 ± 0.067	2.016 ± 0.203	1.020	1.914 ± 0.011	1.866 ± 0.153	0.9749
**Fe–P2**	1.979 ± 0.061	2.032 ± 0.236	1.027	1.891 ± 0.031	1.873 ± 0.007	0.9905
**Co–P1**	1.974 ± 0.053	2.019 ± 0.208	1.022	1.894 ± 0.008	1.872 ± 0.120	0.9884
**Co–P2**	1.974 ± 0.058	2.033 ± 0.234	1.030	1.879 ± 0.017	1.875 ± 0.003	0.9979
**La–P1**	1.974 ± 0.059	2.032 ± 0.226	1.029	–	–	–
**La–P2**	1.982 ± 0.069	2.033 ± 0.236	1.026	–	–	–

Table S3. Average in-plane (d′) and out-of-plane (d″) metal–oxygen bond lengths, together with their ratio, for Ti-centered and Co/Fe-centered (M) octahedra in pristine and doped $Bi_4Ti_3O_{12}$ structures shown in Figure 12 of the main text. All distances are given in Å.

S4. Selection of the Hubbard U Parameter

U values in GGA+U calculations are generally chosen on a semi-empirical basis, often guided by experimental observables such as the band gap; however, inappropriate values may introduce spurious electronic features associated with the corrected orbitals.[34] The choice of the Hubbard parameters for Fe and Co in the present work was therefore evaluated base on a inspection of the band structures obtained across a series of trial U values (Figure S2), and by direct comparison with hybrid-functional (HSE06) band structures (Figure S3).

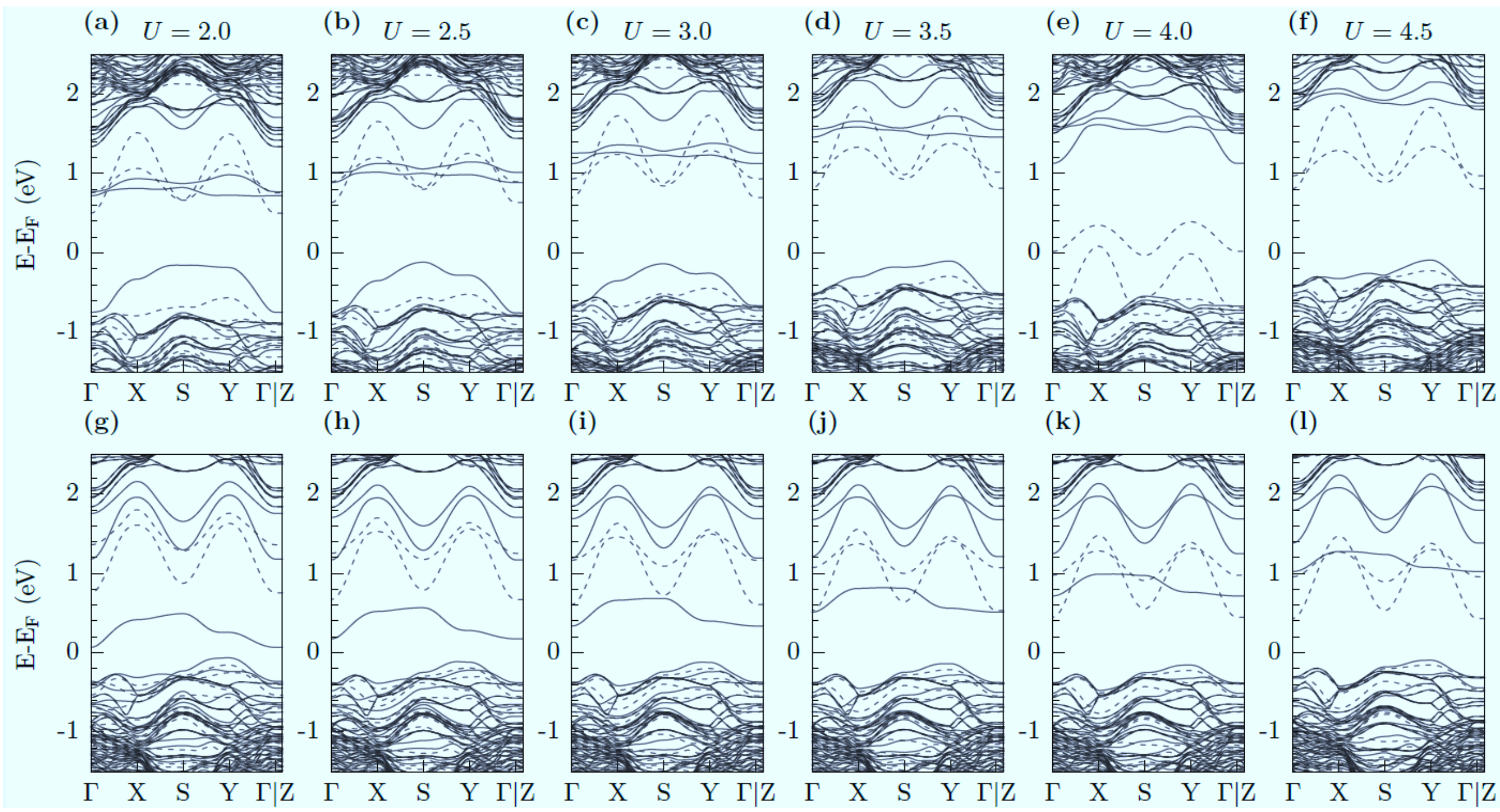

Figure S2. Spin-resolved band structures for the Fe-doped (a–f) and Co-doped (g–l) aurivillius structure calculated with different Hubbard U values applied to the Fe-3d and Co-3d orbitals. All band structures are aligned with the Fermi level. Solid and dashed lines denote the spin-up and spin-down channels.

Comparison with the hybrid-functional band structures (Figure S3) indicates that the GGA+U results obtained with $U_{Fe} = 3.5$ eV and $U_{Co} = 3.0$ eV most closely reproduce the qualitative features of the valence- and conduction-band regions among the trial values tested. Although these parameters do not reproduce the experimental band gap quantitatively, as is typical of semilocal DFT+U approaches, they provide the most balanced description of the electronic structure of the doped systems and were therefore adopted for all calculations reported in the main text.

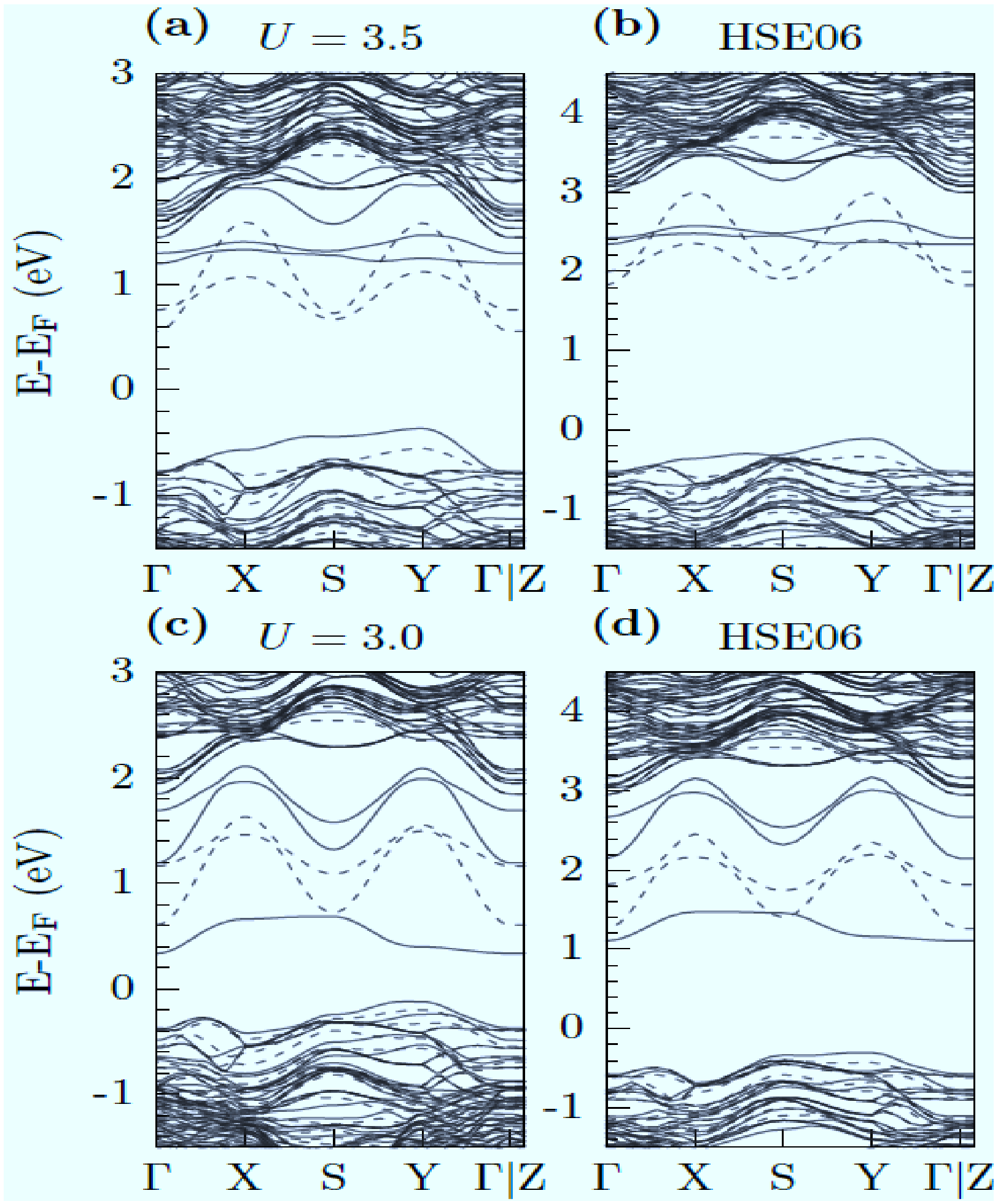

Figure S3. Comparison between the selected GGA+U and hybrid-functional HSE06 band structures for the doped systems. Panels (a) and (b) correspond to the Fe-doped system calculated with U_{Fe} = 3.5 eV and with the hybrid functional, respectively, whereas panels (c) and (d) show the corresponding comparison for the Co-doped system using U_{Co} = 3.0 eV. All band structures are aligned with the Fermi level.

Supplementary References

(78) Crystallography Open Database. Information card for entry 1534182: $Bi_4Ti_3O_{12}$, 2015.

Note: reference numbers above continue the numbering of the main manuscript reference list (1–77).
